# Anisotropy of Crumbs and aPKC Drives Myosin Cable Assembly during Tube Formation

**DOI:** 10.1016/j.devcel.2012.09.013

**Published:** 2012-11-13

**Authors:** Katja Röper

**Affiliations:** 1 MRC-Laboratory of Molecular Biology, Cambridge CB2 0QH, UK

## Abstract

The formation of tubular structures from epithelial sheets is a key process of organ formation in all animals, but the cytoskeletal rearrangements that cause the cell shape changes that drive tubulogenesis are not well understood. Using live imaging and super-resolution microscopy to analyze the tubulogenesis of the *Drosophila* salivary glands, I find that an anisotropic plasma membrane distribution of the protein Crumbs, mediated by its large extracellular domain, determines the subcellular localization of a supracellular actomyosin cable in the cells at the placode border, with myosin II accumulating at edges where Crumbs is lowest. Laser ablation shows that the cable is under increased tension, implying an active involvement in the invagination process. Crumbs anisotropy leads to anisotropic distribution of aPKC, which in turn can negatively regulate Rok, thus preventing the formation of a cable where Crumbs and aPKC are localized.

## Graphical abstract

**Graphical Abstract:**

**Figure:**
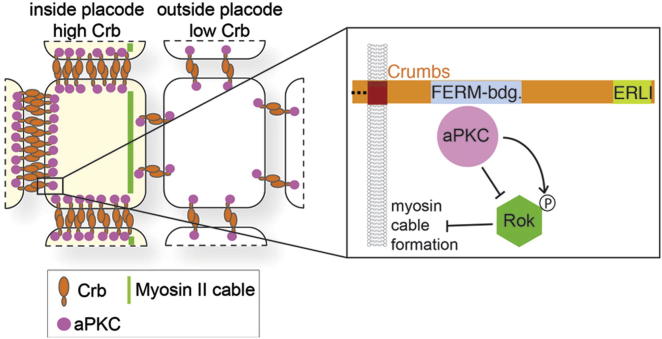

**Highlights:**

► A circumferential actomyosin cable exerts centripetal tension during tube formation ► Anisotropic localization of Crumbs directs actomyosin cable assembly ► Crumbs anisotropy is driven by homophilic interactions of its extracellular domain ► Crumbs directs aPKC anisotropy to negatively regulate myosin II, probably through Rok

## Teaser

Formation of tubular structures from epithelial sheets is a key process of organ formation in all animals. Röper shows that anisotropic localization of Crumbs, mediated by homophilic interactions of its extracellular domains, positions a myosin cable that is part of the machinery driving tube formation in the *Drosophila* embryo.

## Introduction

Cell shape changes and rearrangements within epithelial tissues provide two of the main mechanisms of organ formation during development. Forces need to be generated within the tissue to drive these changes. Although the specification of groups of cells that are fated to become a particular tissue has been well elucidated over the last years, the detailed mechanisms that govern the generation of forces within tissues and cells, and their translation into coordinated shape changes, are not well understood. The cytoskeleton is the most likely source of force generation within tissues, because of the polymerization dynamics of actin and microtubules and also the inherent contractility of actin-myosin networks. Nonmuscle myosin II-actin networks have been found to direct some concerted epithelial rearrangements (for reviews see Kasza and Zallen, 2011; Lecuit and Lenne, 2007): cells within epithelial sheets that undergo convergent extension movements exhibit planar localized myosin II activity during the shrinking of selective cell boundaries to allow neighbor exchanges (Bertet et al., 2004; Simões Sde et al., 2010); during mesoderm invagination in *Drosophila*, ventral furrow cells accumulate myosin II in an apical medial web during the constriction of apical surfaces that drives tissue folding (Martin et al., 2009, 2010). Myosin has the ability to form many different dynamic structures within a cell, but an understanding of myosin II regulation that leads to the selective formation of specific actin-myosin structures in particular processes is lacking in most cases.

**Figure 1:**
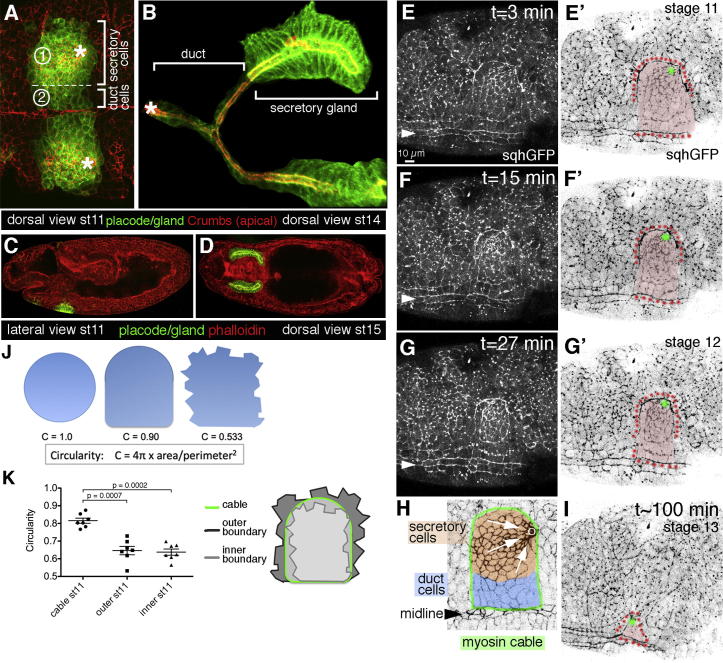
A Prominent Myosin II Cable Surrounds the Salivary Gland Placode during Tube Formation (A–D) Illustration of salivary gland morphogenesis. Red: Crumbs outlining apical cortices (A and B), phalloidin labeling F-actin (C and D); green: *UAS-membrane-GFP* specifically expressed in the salivary glands using *fkh-Gal4* (Maybeck and Röper, 2009). (A) Ventral view of the two salivary gland placodes at stage 11. Brackets: position of the cells fated to form the secretory part of (1) the gland and (2) the duct; asterisks: focal points through which the cells invaginate. (B) Projection of a confocal stack showing the fully formed gland at stage 14. Brackets: formed secretory and ductal parts; asterisk: opening of the duct on the surface of the embryo. (C and D) Confocal sections showing position of glands in whole embryos (C, lateral view; D, dorsal view). (E–G) Still pictures of a time-lapse movie (see Movie S1) of a representative *sqh*^*AX3*^*; sqh::sqhGFP42* (*sqhGFP*) embryo (Royou et al., 2004). Images are a projection of a confocal z stack covering the apical surfaces of the placodal cells and the surrounding tissue. Arrowheads: ventral midline. (E′–G′) Inverse color panels of (E–G). (H) Schematic illustrating the area that is shown in (E–G′) and (I). (I) Inverse color panel of a later time point when the invagination hole has moved toward the ventral midline. In all inverse panels, myosin II cable (red dots) and the invagination point (green dots) are highlighted. (J and K) Analysis of circularity of the salivary gland placode boundary as a measure of smoothness and tension. (J) The circularity of a perfect circle is C = 1 = 4π x area/perimeter^2^ (left). The circularity of the idealized outline of the placode is close to this value (C = 0.9; middle), whereas the circularity of a random outline of cell boundaries is not (C = 0.533; right). The circularity of the placode boundary where the cable is located was significantly greater than that of the boundaries shifted inside the placode by one cell (inner boundary) or outside the placode by one cell (outer placode; see schematic in K). n = 7; represented as mean ± SEM; p values are indicated in the panel. See also Movie S1.

Many organs in both vertebrates and invertebrates are tubular in structure and are formed through processes collectively termed tubulogenesis, often starting from epithelial sheets (Andrew and Ewald, 2010). The *Drosophila* salivary glands are a paired tubular organ that forms during embryogenesis from a primordium of specified epidermal cells called the salivary gland placode (Figures 1A and 1C). Once specified, placodal cells cease dividing and begin to internalize in an ordered fashion, starting with the cells in the dorsoventral corner of the placode, leading to the formation of two secretory glands connected by a Y-shaped duct by the end of embryogenesis (Figures 1A–1D). As the secretory cells do not divide or undergo apoptosis during invagination, the early tissue bending and invagination is driven solely by shape changes and rearrangements of the placodal cells. Embryos zygotically mutant for myosin II (and thus with only reduced levels of myosin) show aberrant invagination of glands (Blake et al., 1998). An increase in apical myosin II has been reported during invagination of salivary glands, but the mode of myosin function is as yet unknown (Escudero et al., 2007; Nikolaidou and Barrett, 2004; Xu et al., 2008).

To elucidate the mechanism by which myosin II is functioning during salivary gland invagination, I performed time-lapse and super-resolution microscopic analysis of embryos expressing a GFP-tagged version of the regulatory light chain of myosin II (MRLC). The live analysis not only revealed strong apical accumulation of myosin II in all cells about to undergo invagination but also revealed a prominent myosin II cable surrounding the salivary gland placode. This cable was under tension and constricted concomitant with the invagination process, indicative of an active involvement in driving the invagination. A step change in Crumbs protein levels between the outermost placodal cells (high levels) and the surrounding epithelial cells (low levels) resulted in a highly anisotropic distribution of Crumbs within the cells at the placode boundary, likely mediated through homophilic interactions between Crumbs extracellular domains. This anisotropic localization of Crumbs was necessary and sufficient for the assembly of the myosin cable. Crumbs acted through induction of anisotropic aPKC localization, with aPKC impinging negatively on Rho-kinase (Ishiuchi and Takeichi, 2011), resulting in an increase in Rok at the placode boundary leading to increased myosin association with the cortex.

## Results

### A Prominent Myosin II Cable Surrounds the Salivary Gland Placode during Tubulogenesis

**Figure 2:**
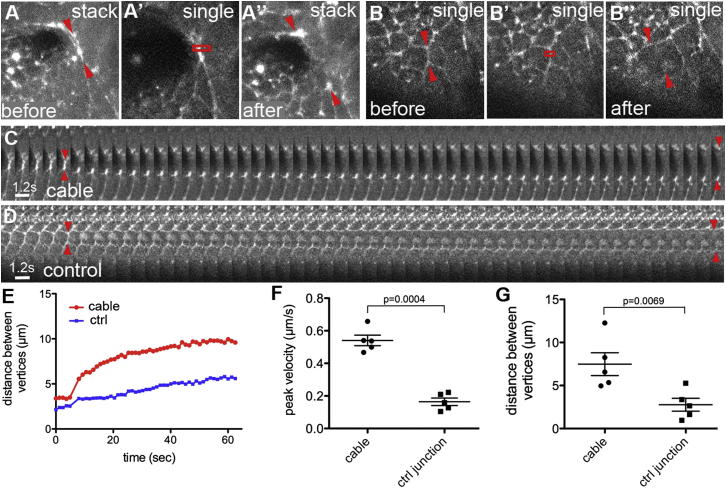
Laser Ablation of the Myosin Cable Reveals It to Be under Increased Tension Using *sqhGFP* embryos, cell edges that form part of myosin cables and control edges enriched in myosin II were ablated. (A) Representative cut in a myosin cable before (A) and after (A″) ablation. (B) Representative cut in a control edge before (B) and after (B″) ablation. Arrowheads indicate vertices that were tracked to determine relaxation dynamics; the boxes in (A′) and (B′) indicate the ablated regions. (C and D) show the matching kymographs for (A–A″) and (B–B″), respectively, of the time-lapse movies taken during edge ablation within the cable (C) (see Movie S2) and in a control edge (D) (see Movie S3). Arrowheads: positions of vertices that were traced at the moment of ablation and of the last time point of the movies. (E–G) Quantification of laser ablation experiments (see Figure S1 for a map of approximate positions of cuts). (E) Example of the faster and greater increase in distance between vertices linked to an ablated edge within a myosin cable (red) compared to the increase in distance for an ablated myosin-rich control edge (blue). (F) The peak retraction velocities were greater for edges within the myosin cable than for control edges (n = 5; represented as mean ± SEM; p = 0.0004). (G) The maximum retraction distances were also greater for edges within the myosin cable than for control edges (n = 5; represented as mean ± SEM; p = 0.0069). See also Figure S1 and Movies S2 and S3.

**Figure 3:**
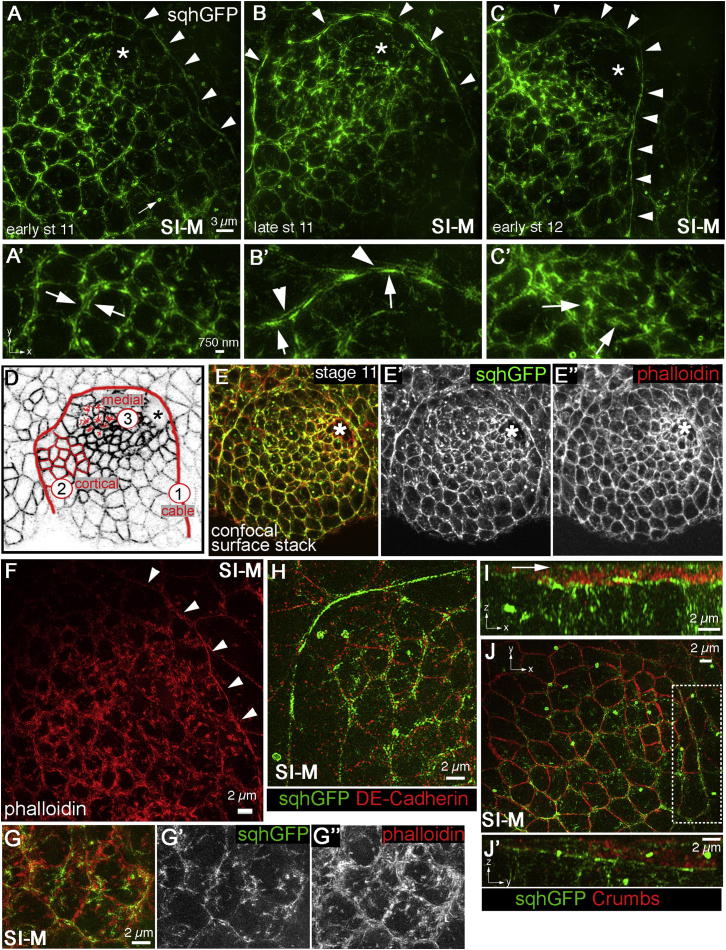
Super-Resolution Microscopy Reveals Distinct Pools of Myosin II during Tube Formation Super-resolution analysis of myosin II localization in the placode using structured illumination microscopy (SI-M). All xy-images labeled SI-M show projections of reconstructed volumes covering the apical 6 μm of the placodal cells. (A–C) From early stage 11 onward, myosin II levels are upregulated in the placodal cells compared to neighboring epithelial cells and the myosin II cable (A–C, white arrowheads), as well as apical cortical and apical medial myosin II can be identified. Labeling is *sqhGFP*. (A and A′) Apical cortical myosin II localized adjacent to the adherens junctions is found as thick layers close to the cell membrane (arrows in A′; see also H below). (B and B′) The apical cortical myosin II forming the cable is especially strong in the placodal cells that contribute to the cable (B′, arrows) compared to the surrounding cells (D′, arrowheads). (C and C′) Dense accumulations of apical medial myosin II in placodal cells close to the invagination hole (C′, arrows). Myosin II fibers often stretch from apical medial clusters toward the apical cortical myosin II. (D) Schematic illustrating the localization of cable (1), and examples of apical junctional (2) and apical medial (3) actomyosin within the placode (at late stage 11). (E–E″) Projection of a confocal z stack at early stage 12, *sqhGFP* (green), phalloidin labeling actin (red). Apical junctional and apical medial actomyosin are visible. (F) Super-resolution SI-M analysis of F-actin localization (labeled with phalloidin) shows a strong enrichment in the placode compared to the surrounding cells (top right corner). Like myosin II, F-actin is found in the cable (arrowheads) and as strong apical cortical and apical medial enrichment in the cells of the placode. (G) Myosin II (G′) and F-actin (G″) colocalize extensively, though not completely, with F-actin, showing a more extended network. (H) Comparison of myosin II and DE-cadherin localization (*sqhGFP* in green and DE-cadherin in red; spots of E-cadherin staining visible in the projection are located within the basolateral membranes of the cells and might represent spot adherens junctions). Apical junctional myosin II is closely flanking the adherens junctions labeled by DE-cadherin (see Movie S4). (I and J) Comparison of myosin II and the apical complex protein Crumbs (*sqhGFP* in green and Crumbs in red). (I) is a cross-section in xz-direction in the middle of the placode; note that *sqhGFP* in green is just below and abutting Crumbs in red. (J) Surface projection of Crumbs and myosin II. (J′) is a cross-section of cells contributing to the cable in yz-direction, with the area marked by a dotted box in (J) being projected. Asterisks indicate the position of the invagination hole. Note that small bright doughnut-shaped objects displayed in the *sqhGFP* channels are remnants of cleavage furrows (see small arrow in A and an artifact of the transgene that does not affect its ability to completely rescue the null mutant (Royou et al., 2004). See also Movie S4.

**Figure 4:**
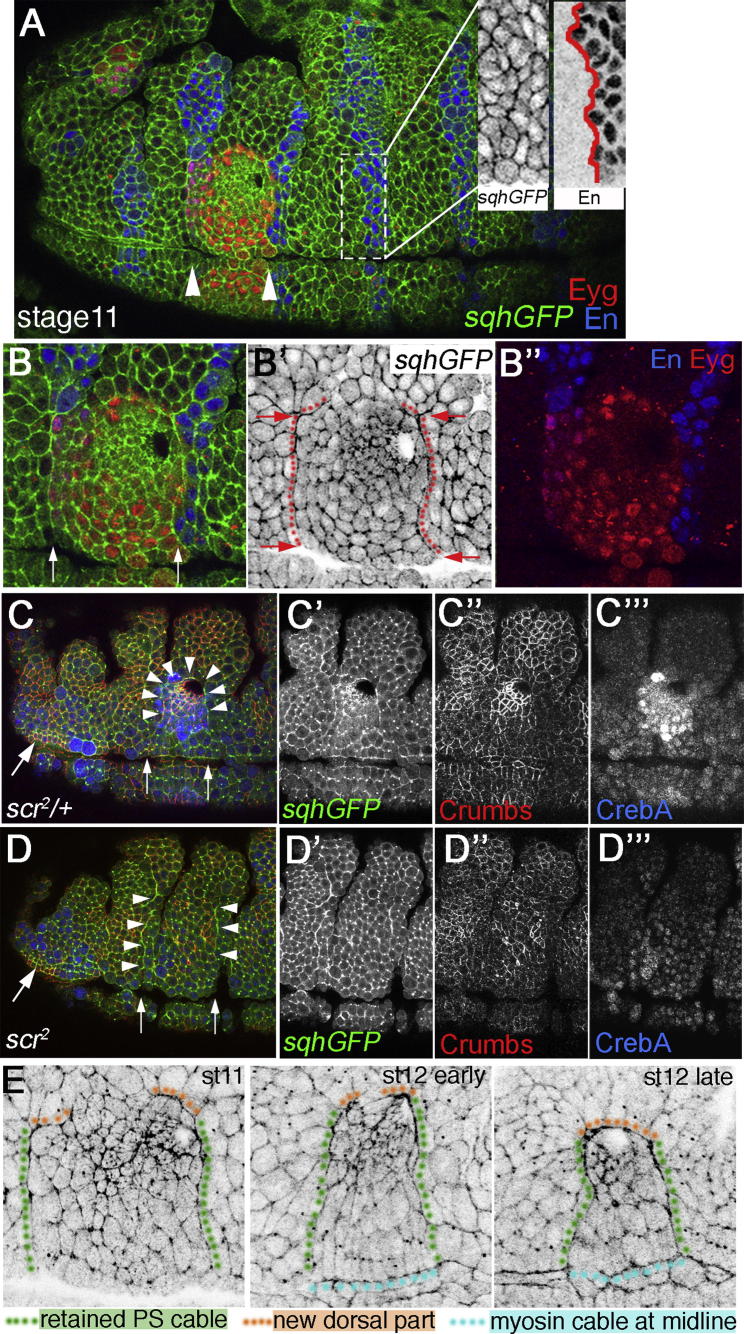
The Actomyosin Cable Incorporates Parts of Parasegmental Boundary Cables (A) A stage 11 wild-type embryo, with the placodal cells that are situated in parasegment 2 (white arrowheads indicate the boundaries of this parasegment) highlighted by Eyegone labeling (Eyg, red); the parasegmental boundaries are highlighted by Engrailed (En, blue; the anterior border of En delineates the parasegmental boundary) and myosin II highlighted by *sqhGFP* (green). Insets in (A): individual myosin II and En channels for the area indicated by a white dotted line. This parasegmental boundary at the posterior end of parasegment 3 is highly irregular at the level of the apical surfaces of cells (highlighted as a red line) and has no myosin II cable present. (B–B″) Higher magnification of the placode area from (A). Only the boundaries at the anterior and posterior border of parasegment 2 (indicated by arrows) have retained a myosin II cable and show highly aligned and straight apical boundaries (B′, red lines and arrows). (C and D) Whereas in the wild-type (C–C″′) the parts of the parasegmental myosin II cables are retained and become connected to a newly forming dorsal section of the cable to surround the whole placode, in a *Scr*^*2*^ mutant, where placodal identity is not specified, the parasegmental cables next to parasegment 2 are often retained (arrowheads in D), but no circumferential cable is assembled. Arrows in (C) and (D) mark the boundaries of parasegment 2. *sqhGFP* (green: C and D; single in C′ and D′); Crumbs (red: C and D; single C″ and D″); CrebA (marking placodal cells, blue: C and D; single in C″′ and D″′). (E) Schematic of the formation of the circumferential cable: remnants of parasegmental boundary cables (green) become connected through a newly forming dorsal section of the cable (orange). The cables at the anterior and posterior sides of the placode are ventrally connected to a myosin cable running along the ventral midline (blue), thus a cable that surrounds the whole placode is formed. See also Figure S2.

Myosin II in the embryo can be visualized using a GFP-tagged MRLC, termed Spaghetti squash (Sqh) in flies, in a *sqh* null mutant background (Karess et al., 1991; Royou et al., 2004). Employing this genetic tool (hereafter called *sqhGFP*), I analyzed the early stages of salivary gland tubulogenesis: the bending of the flat epithelial sheet of the epidermis to form a dimple and then an invaginating tube. Myosin II showed a very striking distribution: in addition to a general apical enrichment in all placodal cells, I observed a myosin II cable that encircled the whole placode (Figures 1E–1H, 3, and 4). This myosin II cable shortened in parallel with the invagination of the placodal cells through a focal point at the dorsal-posterior of the placode (Figures 1E–1I; Movie S1 available online). This process was reminiscent of myosin II cables or “purse-strings,” containing a contractile array of actin and myosin II, observed previously during wound closure in both flies and vertebrates (Martin and Parkhurst, 2004) and also during dorsal closure in fly embryos (Franke et al., 2005). The border of the placode where the cable was observed was very smooth at all stages of invagination: the circularity of the placode boundary was significantly higher than that of a traced line following either one cell boundary further inside the placode or one further outside the placode (for a detailed explanation see the figure legends of Figures 1J and 1K; see also the Experimental Procedures). The observed smoothening suggested that the cable was under tension. To test this directly, the myosin II cable at the placode boundary was disrupted using laser ablation. Using *sqhGFP* embryos, a two-photon laser tuned to 730 nm was focused on a submicrometer section of the cable in order to sever it and the adjacent cell edge. This led to a rapid retraction of the myosin II from the cut site and relaxation of vertices closest to the cut (Figures 2A–2A′ and 2C; Movie S2). The initial peak velocity of the retraction of individual vertices flanking the cut edge is a measure of the tension the ablated edge was under just prior to the cut (Rauzi et al., 2008), as is the maximum displacement of vertices adjacent to the ablated edge (Fernandez-Gonzalez et al., 2009). Ablations of myosin II within the cable were compared to ablations of myosin II-rich boundaries either in the central region of the placode or within the epidermis outside the placode (Figures 2B–2B″, 2D, and S1E; Movie S3). The mean peak retraction velocity of linked vertices after cuts in the myosin cable was 0.54 (± 0.03) μm/s, more than three times higher than the velocity of vertices linked to control edges with 0.16 (± 0.02) μm/s (Figure 2F). In addition, the maximum displacement of vertices of edges that were part of the myosin cable was 2.7 times higher than of control vertices (7.48 ± 1.33 μm versus 2.77 ± 0.75 μm, respectively; Figure 2G). These data demonstrate that the myosin cable is under increased tension, thus generating a centripetal force that is impinging on the cells within the placode.

### Cortical, Medial, and Purse-String Actomyosin Are Abundant during Gland Tubulogenesis

To gain a more detailed view of myosin II localization in the placodal cells, I employed super-resolution microscopy based on structured illumination (SI), which has a resolution of ∼120 nm in xy-plane (Schermelleh et al., 2008). SI microscopy revealed that myosin II was not only organized into a cable bordering the placode but was also present near the apical cortex at the level of adherens junctions and in an apical medial web (Figures 3A–3C′ and schematic in Figure 3D). The myosin II cable at the border appeared to be assembled by the cells on the inside of the placode, as the intensity of myosin II on the inside was stronger, whereas the outside cells only showed normal levels of junctional myosin II (Figure 3B′, compare arrows to arrowheads). Apical cortical myosin II in cells in the center of the placode was clearly composed of dense myosin II accumulations in both cells on either side of a junction (Figures 3A′ and 3H), and apical medial myosin II appeared as a dense network in the center of a cell with finer connections to apical junctional areas (Figure 3C′). To form a contractile array, myosin II has to interact with F-actin, and so phalloidin labeling was compared to myosin II. Similar to myosin II distribution, confocal and SI microscopy showed F-actin within the placode to be enriched in the cable (Figures 3E–3E″ and 3F), at apical junctional areas and in a dense medial apical mesh colocalizing largely with myosin II (Figures 3G–3G″).

When myosin II was compared to membrane markers, such as the apical complex constituent Crumbs (Bulgakova and Knust, 2009) and the junctional protein E-cadherin using SI-microscopy, the dense apical junctional myosin II was found to be located just basal to and abutting the domain of Crumbs localization (Figures 3H–3J′) and to flank the junctional area marked by E-cadherin (Figure 3H; Movie S2), whereas apical medial myosin II was apical to both cadherin and Crumbs (Figure 3I, arrow).

This distribution of myosin II and actin indicated that different pools of actomyosin worked in a coordinated fashion during tube formation.

### Formation of the Cable Is Based on Specifically Retained Cables at Two Parasegmental Boundaries

To understand how and when the supracellular actomyosin cable was formed around the placode, I analyzed myosin II localization with respect to Eyegone (Eyg), a marker of placode fate (Aldaz et al. 2003), and Engrailed (En), a marker of parasegmental organization of the embryo (Figures 4A–4B″). The placode is entirely contained within parasegment 2 (Andrew et al., 1994), and thus the parts of the cable at the lateral sides of the placode border coincided with the parasegmental boundaries. In the embryo, up until the end of stage 10, myosin II can be found arranged in supracellular cables at this parasegmental boundaries that constitute compartment boundaries, where the cables assist in preventing cell mixing upon division (Monier et al., 2010). These myosin II cables had disappeared from most parasegmental boundaries by early stage 11, when the salivary gland placode is first specified (Figure 4A, insets). Only the ventral parts of the two cables flanking parasegment 2, and thus the salivary gland placode, were still present (Figures 4A–4B″; see red lines and arrows in Figure 4B′; see also schematic in Figure 4E). These retained cables alone could not account for the circumferential cable observed at later stages, and indeed they became connected to form the circumferential cable during stages 11–12 through a newly forming section of the cable at the dorsal border of the placode (see schematic in Figure 4E; see also the first three time points of Movie S1). Evidence to support that the rearrangement into a circumferential cable is part of the program driving salivary gland formation came from examining a mutant in *Sex combs reduced* (*Scr*), which encodes the homeotic transcription factor Scr that is necessary and sufficient (within parasegment 2 and anterior to it) for the induction of salivary gland fate (Andrew et al., 1994; Henderson and Andrew, 2000). In *Scr* mutant embryos the two myosin II cables at the boundaries flanking parasegment 2 were often retained but remained unconnected dorsally at a stage when in the wild-type more than 50% of secretory cells have invaginated from the placode and the circumferential cable was clearly established (compare Figures 4C–4C″ and 4D–4D″).

These data show that a newly forming dorsal part of the myosin cable (Figure 4E, orange) was required to join together retained parts of earlier parasegmental myosin II cables (Figure 4E, green). As the myosin cable around the placode was ventrally linked to a myosin cable running along the ventral midline (Figure 4E, blue), this resulted in the circumferential actomyosin cable encircling the placode and exerting centripetal force.

### The Membrane Protein Crumbs Displays Anisotropic Localization, Complementary to Myosin Cable Localization, in Cells at the Placode Boundary

The existence of the actomyosin cable raised the question as to how it was located specifically around the placodal cells. In other developmental systems, accumulation of actomyosin into supracellular structures can involve a number of upstream factors, including differing levels of the adhesion protein Echinoid, cadherin, or the PDZ protein Bazooka or EGF-R signaling (Bertet et al., 2004; Major and Irvine, 2006; Nishimura et al., 2007; Simões Sde et al., 2010), but neither of these appeared to show a localization pattern suggestive of involvement in establishing the cable around the salivary gland placode (see Figure S2). Instead, a possible candidate to play this role in the salivary glands was the plasma membrane protein Crumbs, a component of the apical complex important for the establishment and maintenance of epithelial polarity in the *Drosophila* primary epithelia (Bulgakova and Knust, 2009). Crumbs is highly enriched in the presumptive secretory cells of the salivary gland placode, and this enrichment precedes any changes in cell shape (Myat and Andrew, 2002). The enrichment is downstream of Scr activity, as it was absent in the *Scr* mutant (Figure 4D), and ectopic Crumbs accumulation could be induced anterior to parasegment 2 by heat-shock-induced overexpression of Scr (see Figure S3). Thus, Crumbs protein levels show a steep step change between placodal cells and the surrounding epidermis, and hence it seemed a good candidate to confer positional information at the border of the placode.

**Figure 5:**
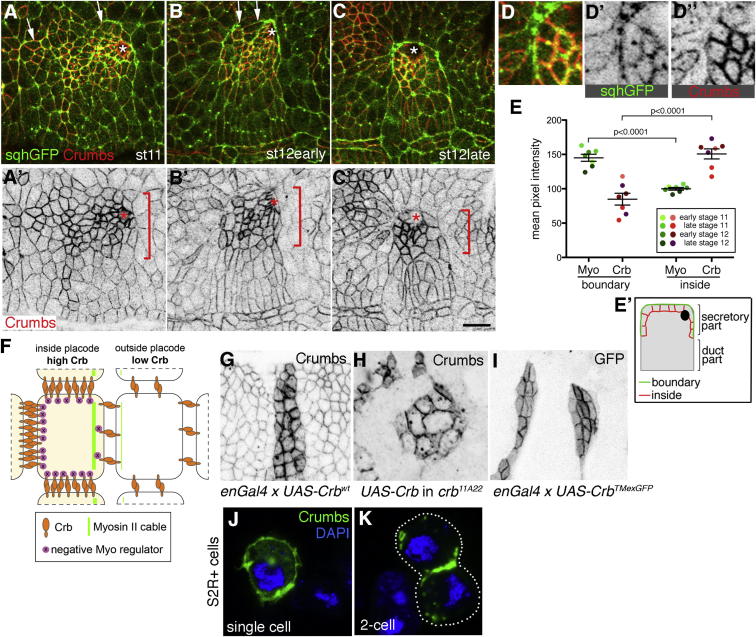
Crumbs Shows an Anisotropic Subcellular Localization at the Border of the Placode that Is Complementary to the Myosin Cable Localization (A–C) Myosin II and Crumbs show highly complementary enrichment in the cells at the border of the placode, increasing from early stage 11 to late stage 12. *sqhGFP* (green, A–C); Crumbs (red, A–C), and single inverse channel in (A′–C′). White and red asterisks indicate the position of the invagination hole. Arrows in (A and B) indicate the extent of the new section of the cable forming dorsally. Crumbs anisotropy and complementarity to myosin II is strongest in the dorsal cells of the placode where the new dorsal section of the cable is forming (red brackets in A′–C′). (D) Higher magnification of a section of (C). *shqGFP* is shown in (D′), and Crumbs is shown in (D″). (E) Quantification of Crumbs and myosin II anisotropy in cells at the boundary using fluorescence intensity. Each point represents the average mean pixel intensity of 6–12 cells at the placode boundary of embryos of the indicated stages. Both Crumbs and myosin II show significant anisotropy (values represented as mean ± SEM). (E′) indicates the edges measured. (F) Working model: Crumbs anisotropy is caused by Crumbs-Crumbs homophilic interactions. If Crumbs intracellular tails are binding a negative regulator of myosin cable formation, this could lead to selective accumulation of the myosin cable at the boundary depleted of Crumbs intracellular tails. (G–I) Crumbs anisotropy is also observed when a stripe of Crumbs is ectopically expressed in the epidermis using *enGal4* (G), when Crumbs is expressed in small groups of cells in a Crumbs null embryo, *crb*^*11A22*^ (H), and when a version of Crumbs encompassing only the extracellular and transmembrane domains fused to GFP, Crb^TMexGFP^, (I) is expressed. (J and K) When Crumbs is ectopically expressed in S2R+ cells (that do not endogenously express Crumbs), it localizes to the whole plasma membrane in single cells (J) (100%, n = 62) but relocates to contact zones in 2-cell clusters (K) (95%, n = 22). Crumbs (green); DAPI (blue). See also Figure S3.

**Figure 6:**
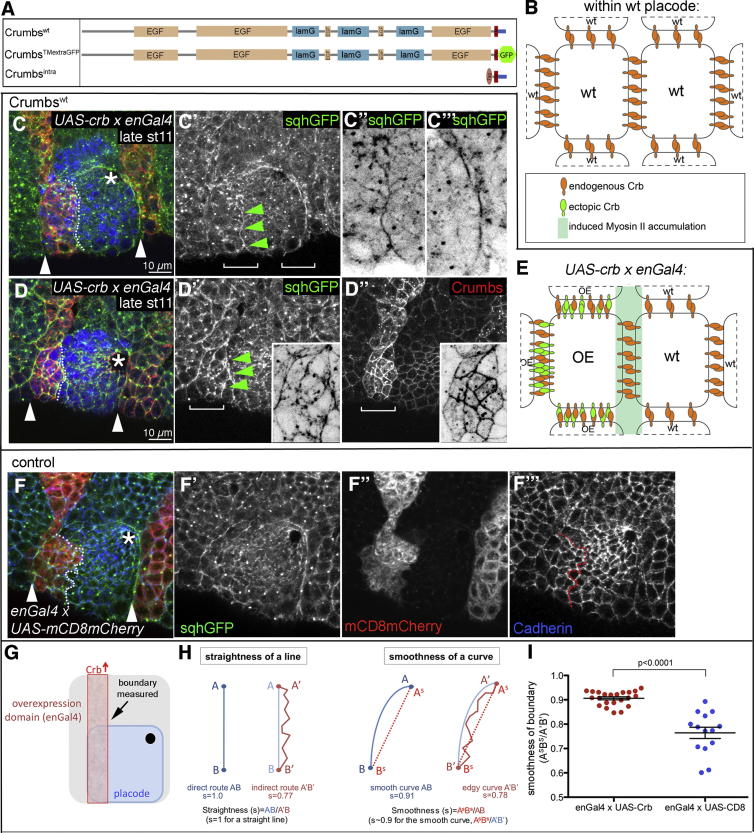
Crumbs Anisotropy Directs the Formation of the Actomyosin Cable at the Placodal Boundary (A) Schematic of Crumbs constructs used for overexpression: wild-type Crumbs (Crumbs^WT^), a version of Crumbs containing only the extracellular and transmembrane domains fused to GFP (Crumbs ^TMextraGFP^), and a version of Crumbs containing only the transmembrane domain and intracellular tail tagged with FLAG (Crumbs^intra^), all under UAS control. (B) Schematic illustrating Crumbs membrane distribution in cells in the center of the placode. (C and D) Overexpression of wild-type Crumbs in an engrailed pattern (using *UAS-Crb*^*WT*^ driven by *en-Gal4*) leads to the accumulation of myosin II at the new boundary of high and low Crumbs levels. Shown are two examples. (C″ and C″′) Inverse panels of magnifications of the ectopically induced cable (C″) and the endogenous posterior cable at the boundary (C″′); positions marked by white brackets in (C′). Inverse insets in (D′ and D″) show magnifications of ectopic and anterior cables (D′) and of Crumbs anisotropy at the edges of the overexpression domain in the placode (D″), indicated by white brackets. At the new boundary, the surface with lower levels of Crumbs is the one with elevated myosin II, as seen at the endogenous placodal boundary. (E) Model: ectopic expression of Crumbs leads to redistribution through homophilic interactions and formation of a new high/low boundary, inducing ectopic accumulation of myosin II at the boundary. (F) Control expression of the membrane protein CD8 tagged with mCherry (*mCD8-mCherry*) using *enGal4* (F″) has no effect on myosin II distribution (F′). *sqhGFP* (green: C, D, F; single in C′–C″′, D′, and F′); Crumbs (red: C, D; single in D″); CrebA (to mark placodal cells, blue C and E); Cadherin (to mark apical boundaries, blue: F; single in F″′). White arrowheads: positions of the endogenous myosin II cables (C, D, and F); green arrowheads: newly induced accumulation of myosin II (C′ and E′). For quantification of ectopic myosin accumulation, see Figure S4. (G–I) Quantification of the smoothness of the newly induced boundary, where the ectopic cable is situated. (G) Position of the ectopic boundary measured (outlined by white dotted lines in C, D, and F). (H) illustrates the here-applied definition of smoothness in comparison to straightness. (I) The ectopic boundary induced by expression of *UAS-Crb*^*WT*^ under *enGal4* control is significantly smoother than control boundaries (*UAS-mCD8mCherry* × *enGal4*). Mean ± SEM are shown; p < 0.0001. See also Figure S4.

Comparison of Crumbs’ subcellular localization to the localization of the actomyosin cable in cells at the border of the placode revealed a highly complementary relationship: Crumbs intensity was highest in the junctions that did not contribute to the actomyosin cable, whereas myosin II was highly enriched only at the junction forming the cable (Figures 5A–5D; see quantification of mean pixel intensities in Figure 5E). The anisotropic and complementary arrangement was most pronounced in secretory cells at the boundary that were forming the cable in the dorsal half of the placode (Figures 5A′–5C′, red brackets). Thus, a change in Crumbs levels did not only demarcate the border of the placode but also the specific absence of Crumbs from the plasma membrane constituting the border in the cells at the border predicted where myosin accumulated. This complementarity between Crumbs and actomyosin cable accumulation could be either coincidental or instructive to the establishment of the cable. If it were instructive, one could propose that Crumbs’ intracellular domain in the cells at the boundary of the placode might actively recruit a negative regulator of myosin cable formation, leaving the plasma membrane at the boundary of the placode free for myosin assembly into a cable (Figure 5F, model). This raises the question of what could mediate Crumbs’ anisotropic localization as the upstream signal? Crumbs has a very large extracellular domain comprising EGF and lamG repeats (see schematic in Figure 6A; Crumbs^WT^), but its role is unclear in most cases in which Crumbs’s function is important (Bulgakova and Knust, 2009). Endogenous Crumbs showed anisotropic localization in the placode, and in addition full-length Crumbs (Crb^WT^; Wodarz et al., 1995) expressed ectopically in a stripe pattern (using Engrailed-Gal4; *en-Gal4*), also led to an anisotropic distribution with more Crumbs accumulating in the contact zones between cells with high expression levels and low levels of Crumbs accumulating where high-expressing cells were in contact with low-expressing cells (Figure 5G). Anisotropic localization could also be observed when small groups of cells expressed Crb^WT^ in an otherwise *crb* null mutant embryo (Figure 5H). Such distribution is reminiscent of the behavior of homophilic adhesion molecules at boundaries between regions of differing expression levels and suggested that Crumbs’ anisotropic distribution could be mediated by homophilic interactions between its extracellular domain (as shown in the model in Figure 5F). In agreement with this, a version of Crumbs containing only the extracellular and transmembrane domains fused to GFP (Crb^TMextraGFP^, see Figure 6A; Pellikka et al., 2002) also showed anisotropic localization when ectopically expressed in stripes (Figure 5I; Figure S4D″), whereas a tagged version containing only the transmembrane domain and intracellular part (Crb^intra^; Klebes and Knust, 2000) showed a homogeneous distribution (Figures S4I and S4I′). In addition, when Crb^WT^ was expressed in fly tissue culture cells that do not express Crumbs endogenously, the protein distributed throughout the plasma membrane in single cells but accumulated at cell-cell contacts in pairs of cells (compare Figures 5J and 5K, respectively).

These data, providing strong evidence of homophilic interaction between Crumbs extracellular domains leading to sorting and rearrangement of Crumbs localization in the plasma membrane of contacting cells in vivo and in vitro, are complemented by recent evidence from zebrafish retinal development (Zou et al., 2012). Together, the zebrafish and fly data point to a conserved fundamental role for homophilic adhesion of Crumbs molecules across species.

### Crumbs Anisotropy Drives Myosin Cable Accumulation

A further question raised by the model proposed in Figure 5F is whether the introduction of a new boundary of high/low Crumbs protein levels would be able to induce formation of a new myosin cable within the placode. Using *en-Gal4*, one stripe of expression overlays the anterior-most part of the placode (see Figures 3A and 3B, blue channel, for the endogenous engrailed pattern and schematic in Figure 6G). In embryos overexpressing Crb^WT^ in this way, the cortices of cells at this new boundary were more aligned and ectopic myosin II accumulated (see Figures 6C–6C″ and 6D–6D″ compared to 6F–6F″; see Figures 6G–6I and Figure S4C for quantifications), presumably because of the introduction of relatively fewer intracellular tails of Crumbs available at the interface of overexpressing and wild-type placodal cells. This suggested that the difference in Crumbs protein levels between the placode border cells and the surrounding corona of epithelial cells with low levels of Crumbs was the trigger that led to Crumbs anisotropy, which in turn induced the localization of the actomyosin cable at this site (see model in Figure 5F). Outside the placode, ectopic Crumbs boundaries did not lead to myosin accumulation at stage 11, but at later stages (st15) myosin accumulation could occasionally be detected (Figures S4A–S4B′). Thus, cells within the placode appeared to be primed to respond to cellular Crumbs anisotropy with actomyosin cable formation at all stages.

When the version of Crumbs containing only the extracellular and transmembrane domains of Crumbs (Crb^TMextraGFP^) was expressed under *en-Gal4* control, an ectopic myosin cable was induced in the placode in about 50% of embryos (Figures S4D–S4D″). This could be due to the titrating away of intracellular tails of Crumbs (and thus negative regulators) from the boundary between overexpressing and wild-type placodal cells, as Crb^TMextra^ would be able to interact with the extracellular domain of Crb^WT^ (see model in Figure S4E, left) or could be caused by negative effects on endogenous Crumbs levels (Figure S4E, left and Figures S5F–S5G′). As described previously, overexpression of Crb^intra^ leads to a drastic effect on endogenous Crumbs, in that it becomes downregulated and is thus absent from the regions of Crb^intra^ expression (Klebes and Knust, 2000). The same effect was observed when Crb^intra^ was expressed in engrailed stripes, with the consequence that the endogenous Crumbs in cells bordering these stripes overexpressing Crb^intra^ now showed a highly anisotropic distribution (Figures S4F″ and S4H). Within the placode, under these conditions, myosin II enrichment was often found at the new border between cells with differing levels of wild-type Crumbs (Figures S4C and S4F–S4F″, green arrowheads in Figure S4F′, and model in Figure S4G).

The importance of Crumbs is also highlighted by the fact that in the *crb* null allele (*crb*^*11A22*^) no myosin II accumulated at all, either within or around the placode, and no invagination could be detected at early stages (Figures S4J–S4K″′). I also analyzed a second, hypomorphic, *crb* mutant, *crb*^*8F105*^, that only lacks the last 23 amino acids of the intracellular domain, thus deleting the PDZ-binding ERLI motif but leaving the FERM-domain binding site of Crumbs intact (see schematic in Figure S4O; Médina et al., 2002). In this mutant, the truncated Crumbs protein and myosin II continued to accumulate in their mutually exclusive anisotropic patterns (Figures S4L–S4M″). Thus, the ERLI domain crucial for most of Crumbs’ function in controlling epithelial polarity is not required for its anisotropic localization or effect on myosin accumulation in the cable. Furthermore, myosin accumulation and cable formation could be rescued to some extent by expressing Crb^WT^ selectively in the placode in otherwise *crb*^*11A22*^ null embryos (Figures S4P–S4P″′). In these embryos, invagination of glands at stage 14 was also drastically improved (Figure S4Q).

Taken together, these data suggest that homophilic interactions between Crumbs’ extracellular domain could determine the local concentration of Crumbs in the plasma membrane that, in turn, lead to the selective, anisotropic formation of a myosin II cable at the salivary gland placode border.

### Crumbs Works through aPKC to Negatively Regulate Rho-Kinase

The Crumbs intracellular tail has been shown to interact with a variety of binding partners (Bulgakova and Knust, 2009). One interacting factor that could mediate the negative regulatory role of the intracellular tail on myosin cable formation is atypical protein kinase C (aPKC). aPKC has been shown to interact directly or indirectly with the Crumbs intracellular domain (Sotillos et al., 2004), and a recent report shows that aPKC can phosphorylate and inhibit Rho-kinase (Rok) in mammalian tissue culture (Ishiuchi and Takeichi, 2011), with Rok being the major activator of myosin light chain.

**Figure 7:**
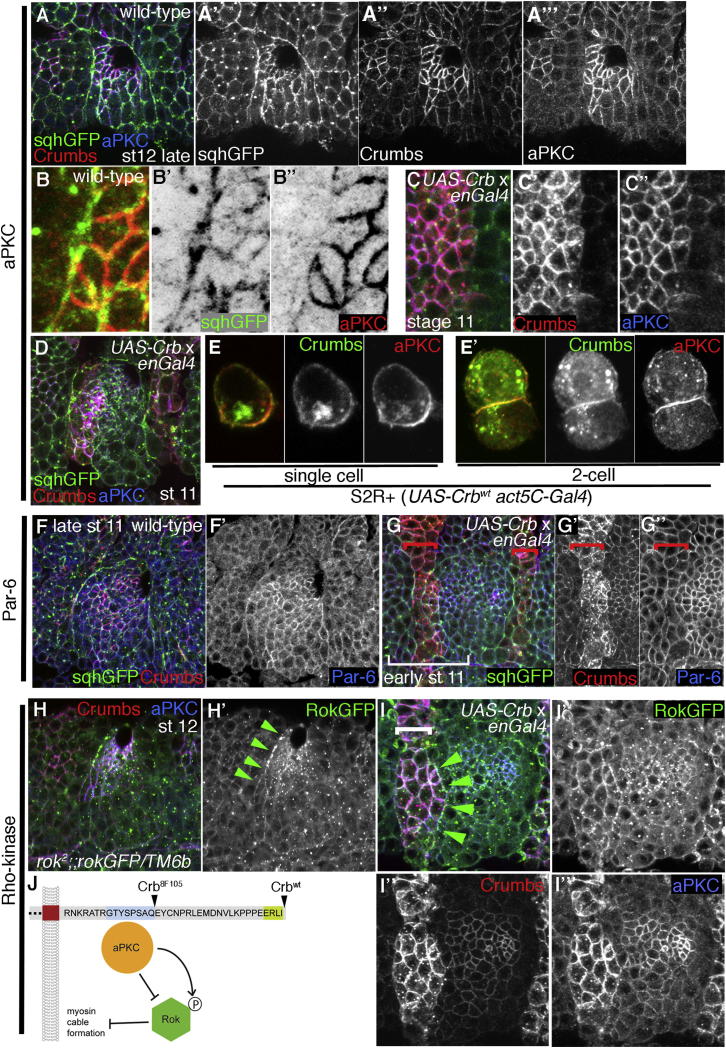
Crumbs Anisotropy Induces aPKC Anisotropy and Affects Rho-Kinase Activity (A and B) aPKC as a candidate negative regulator of myosin cable formation shows anisotropic localization at the placode border; higher magnification shown in (B–B″). (C and D) Ectopically expressed Crumbs (using *UAS-crb*^*WT*^ and *enGal4*) recruits aPKC. Examples of an overexpressing region bordering wild-type cells are shown for stage 11 embryonic epidermis (B–B″) and for stage 11 placode area (D). *SqhGFP* (green: A and B; single in A′ and B′); Crumbs (red A, C, and D; single in A″ and C′); aPKC (red: B; blue: A, C, and D; single in A″′, B″′, and C″). (E) Expression of Crb^WT^ in S2R+ cells that do not endogenously express Crumbs leads to recruitment of aPKC from a cytoplasmic localization (not shown) to the plasma membrane (E) in single cells (100%, n = 50). In two-cell clusters, Crb accumulating at the contact zone between cells also recruits aPKC to this location (E′; 100%, n = 21). Crb (green in E and E′ and as single channel); aPKC (red in E and E′ and as a single channel). (F and G) Par-6, a component of the aPKC complex, is unlikely to mediate aPKC recruitment to Crumbs tails in the placode. (F and F′) Par-6 is enriched in the apical domain of placodal cells but does not show the same anisotropic localization as Crumbs and aPKC (see Figure S5). (G–G″) Expression of *UAS-Crumbs*^*WT*^ in *enGal4* stripes (red brackets) does not redistribute endogenous Par-6 inside and outside the placode at stages 11–12. (G′) and (G″) show the region indicated by a white bracket in (G) (see higher magnification in Figure S5). *SqhGFP* (green: F and G; Crumbs (red: F and G; single in G′); Par-6 (blue: F and G; single in F′ and G″). (H and I) Rok is a candidate target of aPKC to mediate the effect on myosin II. (H and H′) Embryos expressing *Ubi-rok-GFP* in a *rok*^*2*^ mutant background show RokGFP enriched apically in the placode and concentrated in the region where the cable localizes (H′, arrowheads). (I–I″′) Introduction of a new Crb high/low boundary (using *UAS-Crb*^*WT*^ and *enGal4*; white bracket in I indicates stripe of expression) leads to accumulation of RokGFP at the new boundary (five out of eight embryos). RokGFP (green: H and I; single in H′ and I′); Crumbs (red: H and I; single in I″); aPKC (blue: H and I; single in I″′). (J) Model of Crumbs action on myosin cable formation and maintenance around the secretory part of the placode: Crumbs tails recruit aPKC through the FERM-binding domain, leading to phosphorylation and inactivation of Rok, thus preventing increased cortical myosin association where Crumbs tails are at high density. This leaves active Rok at the Crumbs-depleted membrane domain free to recruit membrane-associated myosin II.

If aPKC works downstream of Crumbs, it should show the same anisotropic localization in cells at the placode boundary. Indeed, endogenous aPKC localization was highly anisotropic (Figures 7A–7A″ and 7B–7B″). Furthermore, the aPKC anisotropy was downstream of Crumbs localization as overexpression of Crumbs in engrailed stripes led to a matching increased anisotropic localization of aPKC, both in the placode and elsewhere in the epidermis (Figures 7C–7C″ and 7D) and also in *Drosophila* S2R+ cells (Figures 7E and 7E′). aPKC interacted with the Crumbs intracellular tail through the FERM-binding domain, as it was still localized in an anisotropic fashion in the *crb*^*8F105*^ allele that expresses the shortened form of Crumbs lacking the C-terminal 23 amino acids (Figures S4N–S4N″). The interaction between Crumbs and aPKC was likely to be direct and not mediated by Par-6, another component of the aPKC complex that has been shown to directly bind to Crumbs (Kempkens et al., 2006): endogenous Par-6 did not display any anisotropy at the placode boundary (Figures 7F and 7F′; Figures S5A–S5A″′) and did not relocalize upon Crb^WT^ expression in an engrailed stripe pattern (Figures 7G–7G″; Figures S5B–S5B″′). The effect of introduction of a step change in aPKC levels was analyzed through expression of a membrane-targeted form of aPKC (Sotillos et al., 2004) in an engrailed pattern. aPKC expressed in this way did not localize in an anisotropic fashion, and its expression also led to downregulation of endogenous Crumbs; therefore, the direct effect of aPKC alone on myosin II localization at the placode boundary could not be determined (Figures S5C–S5D″′).

aPKC can negatively regulate Rok (Ishiuchi and Takeichi, 2011), and therefore Rok localization was analyzed. A ubiquitous Rok-GFP transgene was enriched apically in cells in the placode and concentrated where the myosin cable localized (Figures 7H and 7H′). When a new step change of Crumbs levels was introduced in the placode (using *UAS-Crb*^*WT*^ under *enGal4* control) more than half of the embryos analyzed showed ectopic accumulation of Rok-GFP at the new boundary (Figures 7I–7I″′), supporting the model that Crumbs and aPKC anisotropy direct Rok localization and activity (Figure 7J). Also, when HA-Rok was expressed in an engrailed pattern in embryos, the increased levels of Rok within the placode led to increased apical constriction (Figures S5H–S5H″′) and induced increased cortical membrane localization of myosin II (Figures S5H′ and S5I). Increasing Rok throughout the placode using fkh-Gal4 led to aberrant glands with lumen defects at later stages (Figure S5J), indicative of failure to execute the correct program of cell shape changes driving invagination and suggesting that myosin II activation by Rok within the placode is tightly controlled in a temporal and spatial manner. Furthermore, expression of aPKC in an engrailed pattern (using *UAS-aPKC[CAAX}*) led to reduced association of Rok-GFP with the cortex and increased apical surface area (Figures S5K–S5K″), supporting a potential negative effect of aPKC on Rok. In addition, in vitro in *Drosophila* S2R+ cells overexpressed HA-Rok localized to the cell cortex together with phospho-myosin II (Figures S5L and S5M), and these cortical HA-Rok levels, together with cortical phospho-myosin II and actin levels, were strongly increased when aPKC function was inhibited (Figures S5N, S5O, S5R and, S5S).

These data, both in vivo and extrapolated from in vitro studies, support a model by which the anisotropic Crumbs at the placode boundary recruits aPKC to negatively regulate downstream Rok at membranes where no myosin cable should accumulate. Active Rok accumulates in placodal cells at the boundary, where aPKC is absent and could thus direct increased cortical association of myosin II in areas of relative Crumbs depletion, leading to cable formation (Figure 7J).

## Discussion

Myosin II has emerged as a key player in morphogenesis because of its ability to form contractile structures together with F-actin that can directly alter the shapes of cells. Different pools of myosin II within epithelial cells undergoing morphogenesis have been observed, namely apical junctional myosin, apical medial myosin, and in addition myosin organized into supracellular structures termed myosin cables or purse-strings (for a recent review, see Kasza and Zallen, 2011). All three myosin II pools have been shown to be important for epithelial morphogenesis, but how much the activities of the pools depend on each other and how their specific assembly is regulated is much less clear.

Using the formation of the invagination of the salivary glands in the fly embryo as a model allowed me to analyze a morphogenetic process in which all three different pools of myosin are present. Upon specification of the gland placode, myosin II levels are drastically upregulated in the secretory cells of the placode, and myosin accumulates at cortical regions and medially within the apical “dome” of each cell. In addition, a supracellular myosin cable surrounding the placode is formed in a process by which parts of existing structures (remnants of parasegmental cables) are joined together with a newly specified dorsal section of the cable (Figure 3).

### A Myosin Cable Involved in Tubulogenesis

Compared to mesoderm invagination in the fly, a well-studied process that depends on both apical medial and cortical myosin assemblies (Martin et al., 2009), the invagination of the tubes of the salivary gland topologically rather resembles wound healing or dorsal closure processes, as the surrounding epidermis is drawn in from around the placode to cover the patch where cells are invaginating into the embryo (Martin and Parkhurst, 2004).

All three processes have in common that the patch of cells “disappearing” from the plane of the epithelium is surrounded by a contractile actomyosin cable. In contrast to wound healing and dorsal closure, the cable in the case of salivary gland tubulogenesis is assembled within the cells on the inside (Figure 3), whereas it is assembled in the surrounding epithelial cells in the former two instances. Thus, the signal for cable assembly is provided by the “inside” cells in the salivary gland placode.

The laser ablation data presented here clearly demonstrate that the cable around the placode is under increased tension, even when compared to other myosin enriched edges. The tension is in magnitude comparable to the tension determined for shorter supracellular myosin cables observed during germband extension in the fly embryo (Fernandez-Gonzalez et al., 2009). This increased tension indicates active involvement in the invagination process. Previous modeling studies on sea urchin invagination have shown that a contractile apical ring surrounding a placode could be a driving force for invagination (Davidson et al., 1995). Interestingly, upon laser ablation the cable around the salivary gland placode was very quickly repaired (see Figure S1), suggesting a continuous signal to assemble myosin at the outermost surface of the placode. This fast repair precluded laser ablation as a means of probing function of the cable in the invagination in contrast to medial and junctional myosin.

### Anisotropy of Crumbs Directs Subcellular Myosin Arrangement

Crumbs, the transmembrane component of the apical protein complex, shows a very striking anisotropic localization at the border of the placode, that is complementary to the accumulation of myosin II forming the cable. The data presented above strongly support a model whereby Crumbs intracellular tails at cell edges facing toward the inside of the placode recruit aPKC, which can act as a negative regulatory factor impinging on Rok, thus preventing cable assembly at edges containing high levels of Crumbs tails. This leaves active Rok at the cell edges forming the placode boundary, where it acts to recruit myosin into the cable.

Interestingly, only the presence or artificial introduction of cortical anisotropy of Crumbs and downstream aPKC has this effect. The central cells of the placode that are not forming the boundary all show strongly upregulated levels of Crumbs, aPKC, Rok, and myosin II, but in these cells a high density of Crumbs tails does not preclude accumulation of junctional membrane-proximal myosin. Thus, the change in density of Crumbs tails, not the overall concentration, is instructive in this system.

Crumbs has previously been shown to have an effect on salivary gland morphogenesis through a proposed regulation of the apical membrane domain (Kerman et al., 2008; Myat and Andrew, 2002) and has been implicated in tracheal pit invagination through regulation of phospho-Moesin (Letizia et al., 2011). Also, members of the Crumbs polarity complex have been shown to be able to interact with the Par3 (Bazooka)/Par-6/aPKC complex (e.g., Par-6 can bind to Crumbs^intra^; aPKC can phosphorylate Crumbs^intra^; Sotillos et al., 2004). Also, an anticorrelation between localization of Crb and aPKC compared to Lgl and myosin has been described in the denticle belts of the fly epidermis (Kaplan and Tolwinski, 2010). This work now describes a potential link from Crumbs through aPKC to Rok and myosin II, which would link the interaction of two different polarity factors directly with the coordination of morphogenesis through myosin II at a molecular level.

### A Function for the Extracellular Domain of Crumbs?

The large extracellular domain of Crumbs has long posed an enigma with regard to its role. Crumbs’ function in epithelial polarity can mostly be mediated by its intracellular domain (Klebes and Knust, 2000). Only for photoreceptor morphogenesis, the extracellular domain appears required within the fly, though its molecular role is unclear (Richard et al., 2009). The protein domains present in the extracellular domain, namely EGF repeats and lamG domains, are both found in many classical and nonclassical cadherins. Data presented here suggest that Crumbs could be organized in the plasma membrane through homophilic interactions of the extracellular domains between molecules on neighboring cells: Crumbs shows highly anisotropic localization within the wild-type placode but also within wild-type cells bordering a *crumbs* mutant clone (Chen et al., 2010) or in clusters of Crumbs expressing cells in a null mutant embryo and within cells at the edge of an ectopic step change in Crumbs expression levels (e.g., when wild-type Crumbs is either overexpressed or depleted in an engrailed stripe pattern; Figure 5). Also in vitro, Crumbs accumulates at contact zones between expressing cells (Figure 5). The extracellular domain appears the ideal candidate to mediate this anisotropy, which is supported by the following findings: (1) the Crb^TMextra-^GFP shows anisotropic localization at borders with cells not expressing the construct (Figure 5); (2) endogenous Crumbs in wild-type cells is induced to localize in an anisotropic fashion when neighboring cells are depleted of endogenous Crumbs and only express the intracellular domain (Figure S4); and (3) the Crb^intra-^FLAG shows uniform expression in cells (Figure S4). These observations exclude that another transmembrane protein that interacts with the intracellular domain of Crumbs in equal stoichiometry could mediate the anisotropy, though it cannot formally exclude that another extracellular factor might act as an intermediary between two Crumbs extracellular domains. These data are strongly supported by recent evidence from zebrafish, where vertebrate Crumbs isoforms appear to mediate homophilic interactions to promote orderly arrays of photoreceptors (Zou et al., 2012). Also, recent data analyzing the establishment of polarity in the *Drosophila* follicular epithelium suggest a role for cis-interaction of Crumbs molecules within a single cell (Fletcher et al., 2012). Thus, a clear role for the Crumbs extracellular domain in organizing plasma membrane domains through homophilic interactions in *cis* and in *trans* is prominently emerging.

Data presented here describe a link between the transmembrane protein Crumbs and myosin II structures actively engaged in controlling morphogenesis. Crumbs’ ability to interact in *trans* allows the step change in Crumbs levels between placode and surrounding cells to be translated into a subcellular asymmetry, the anisotropic localization of Crumbs. This mechanism provides the cells at the border of the salivary gland placode with the means of sensing this positional information and allows them to turn the positional information into a morphogenetic readout: myosin cable formation. In the future it will be interesting to determine if the arrangement of Crumbs and myosin II described here is conserved during topologically similar processes of tube invagination, such as, for instance, the side budding of branches during lung or mammary gland morphogenesis.

## Experimental Procedures

### Fly Stocks and Husbandry

For a full list of fly stocks used, see the Supplemental Experimental Procedures.

### Embryo Immunofluorescence Labeling, Confocal, Live, and Super-Resolution Analysis

Embryos were stained using standard procedures (see the Supplemental Experimental Procedures, also for details of antibodies used).

For live time-lapse analysis of *sqh*^*AX3*^*; sqh::sqhGFP42* embryos, the embryos were dechorionated in bleach, rinsed in water, and attached to a coverslip with the ventral side up using heptane glue and covered with Halocarbon Oil 270 saturated with water. Time-lapse sequences were acquired on an Olympus FluoView 1000 Confocal Laser scanning system as z stacks. Z stack projections to generate movies were assembled in ImageJ or Imaris.

For three-dimensional (3D) structured illumination microscopy studies (Schermelleh et al., 2008), *sqh*^*AX3*^*; sqh::sqhGFP42* embryos were fixed as previously described and double-labeled with anti-Crumbs, anti-Cadherin (both with Alexa594-coupled secondary antibodies; Invitrogen, Carlsbad, CA, USA) or phallodin-594 (Molecular Probes, Eugene, OR, USA) and mounted in Citifluor (Citifluor Ltd., London, UK). Imaging was performed using the DeltaVision OMX 3D-SIM System (Applied Precision, Issaquah, WA, USA). All data capture used an Olympus 100× 1.4NA oil objective, 488 nm and 593 nm laser illumination and standard excitation and emission filter sets. 3D-SIM images were sectioned using 125 nm z-step size. Raw three-phase images were rendered and reconstructed in 3D by softWoRx 4.5.0 (Applied Precision) software.

### Laser Ablation Experiments

Embryos were prepared as previously described for live time-lapse analysis. Laser cuts were performed on a Zeiss 780 Laser Scanning Microscope equipped with a Chameleon Two-Photon Ti:Sapphire Laser, using the 40×/1.3 Oil DIC M27 Zeiss objective. The Two-Photon laser was tuned to 730 nm. After five precut scans ∼1.2 s apart, the laser ablation was performed with a single scan iteration with 16.81 μsec dwell time per pixel (and 0.09 × 0.09 μm/pi) at 20% laser output. Ablated regions were usually about 7 × 20 pixels in dimension (i.e., about 0.55 × 2.63 μm, oriented to ablate about 0.55 μm of the edge). The ablation was followed by a further 44 scans, each ∼1.2 s apart, by which time maximum relaxation had been achieved in most cases. Vertices were manually tracked in ImageJ, and resulting distances between vertices adjacent to cuts were plotted in Excel and Prism to extract peak retraction velocities and maximum displacement values, indicative of bond tension prior to the cut (Fernandez-Gonzalez et al., 2009; Rauzi et al., 2008). Statistical significance was determined using paired t test.

### Circularity and Smoothness Quantifications

Cell bonds were manually traced in ImageJ to quantify the length and area of the boundary corresponding either to the salivary gland placode boundary or the boundary shifted one cell layer inside or outside the placode boundary. Circularity was calculated based on the fact that for a perfect circle the circularity C = 1 = 4 π area/perimeter^2^ (Lawrence et al., 1999). Circularity values were plotted in Prism and statistical significance of differences in circularity of inner and outer boundaries compared to circularity of the cable analyzed using paired t test.

Please see the Supplemental Experimental Procedures for details of smoothness quantifications.

### Anisotropy Quantifications

User drawn three pixel-wide lines for edges that were part of the boundary/cable or were inside edges of the same cells were used to calculate the mean pixel intensity. Six to twelve representative cells were measured per placode and averaged per embryo. Mean pixel intensity values were plotted in Prism, and statistical significance of differences in intensity between “boundary” and “inside” edges were analyzed using paired t test.

## Supplemental Information

Document S1. Figures S1–S5 and Supplemental Experimental Procedures

Movie S1. A Prominent Myosin II Cable Surrounds the Salivary Gland Placode during Tube Formation, Related to Figure 1 Time-lapse movie of a *sqh*^*AX3*^; *sqh::sqhGFP42* embryo, viewing the area around one placode on the ventral side of the embryo. A still of this movie is shown in Figure 1. The movie is a projection of a confocal z stack to cover all apical myosin II in the area imaged. Length of the movie is 54 min; z stacks were acquired every 3 min.

Movie S2. Laser Ablation of a Cell Edge that Was Part of the Myosin Cable around the Salivary Gland Placode, Related to Figure 2 Time-lapse movie of *sqhGFP*; a single confocal section is shown. Frames are ∼1.2 s apart; the cable is ablated after time point 5 (green arrow appearing to indicate the position of ablation).

Movie S3. Laser Ablation of a Control Cell Edge Enriched in Myosin, Related to Figure 2 Time-lapse movie of *sqhGFP*; a single confocal section is shown. Frames are ∼1.2 s apart; a myosin-rich edge within the placode that is not part of the myosin cable is ablated after time point 5 (green arrow appearing to indicate the position of ablation).

Movie S4. Animated 3D-Volume Rendering of a SI-M Stack Comparing *sqhGFP* and DE-Cadherin, Related to Figure 3 *sqhGFP* is in green and DE-cadherin in red. Scale bar is 2 μm, and the depth of the stack is 6 μm. Note that the bright doughnut-shaped structures strongly labeled by *sqhGFP* are remnants of cleavage furrows that accumulate ectopically in the *sqh*^*AX3*^*;sqh::sqhGFP42* transgenic embryos but that appear to have no effect on *sqhGFP* function in this genetic background (Royou et al., 2004).
