# Supplementary material for: Anisotropy of Crumbs and aPKC Drives Myosin Cable Assembly during Tube Formation
Source: Dev Cell. 2012 Nov 13;23(5):939–53. doi: 10.1016/j.devcel.2012.09.013 (PMC3562440; doi:10.1016/j.devcel.2012.09.013)
Supplement: Document S1. Figures S1–S5 and Supplemental Experimental Procedures [file mmc1.pdf]

Developmental Cell, Volume *23*

Supplemental Information

Anisotropy of Crumbs and aPKC Drives Myosin Cable Assembly during Tube Formation

Katja Röper

Supplemental Information Inventory

Supplemental Figures S1-S5 and Legends,

Supplemental Experimental Procedures

Supplemental References

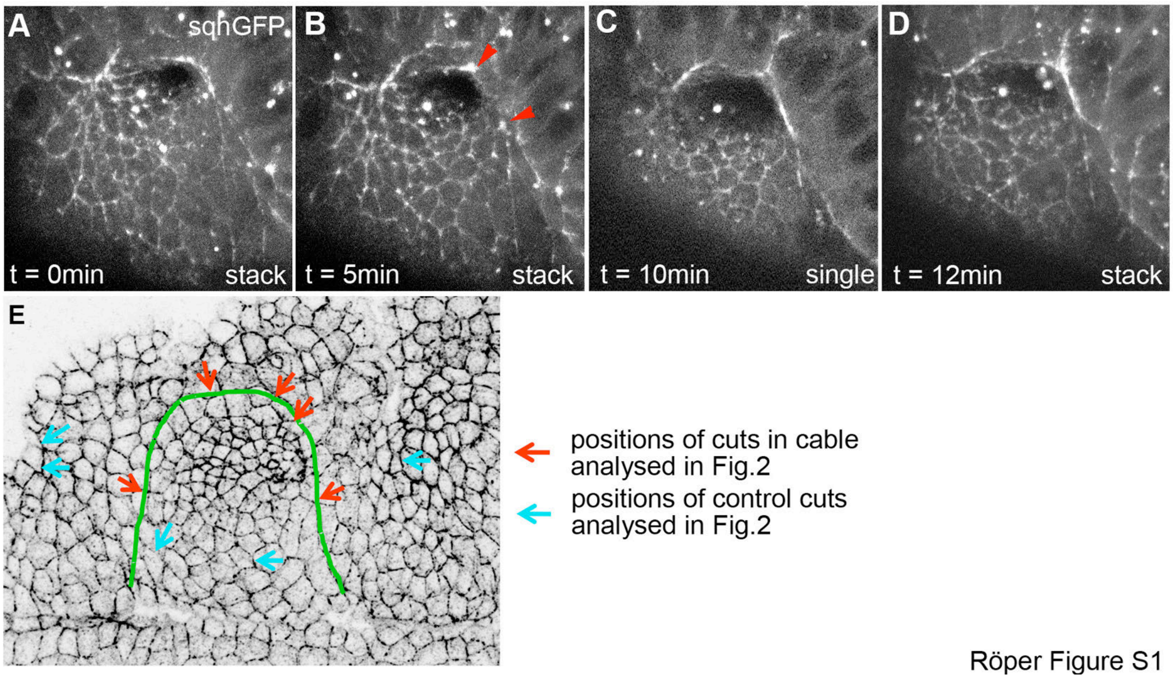

Röper Figure S1

Figure S1, related to Figure 2. Upon laser ablation the myosin cable surrounding the placode is quickly repaired.

A-D Confocal stacks (**A, B, D**) and a single confocal section (**C**) of the area of the myosin cable ablated and analysed in Fig.2A-A'' at the indicated time points: **A** is a stack taken just before laser ablation of the cable, **B** is a stack taken 5 min after ablation, **C** is a single section taken 10 min after ablation, and **D** is a stack taken 12 min after ablation. Arrowheads in **B** indicate the position of the retracted vertices after ablation. Note that after 10 min already, sqhGFP has accumulated again at the previously ablated edge and seems to be incorporated into the remaining cable. This newly repaired section is under tension as it can be ablated again with concomitant recoil of linked vertices (data not shown).

E Map of approximate positions of laser cuts analysed in Fig.2. Red arrows: cuts in the myosin II cable, blue arrows: control cuts.

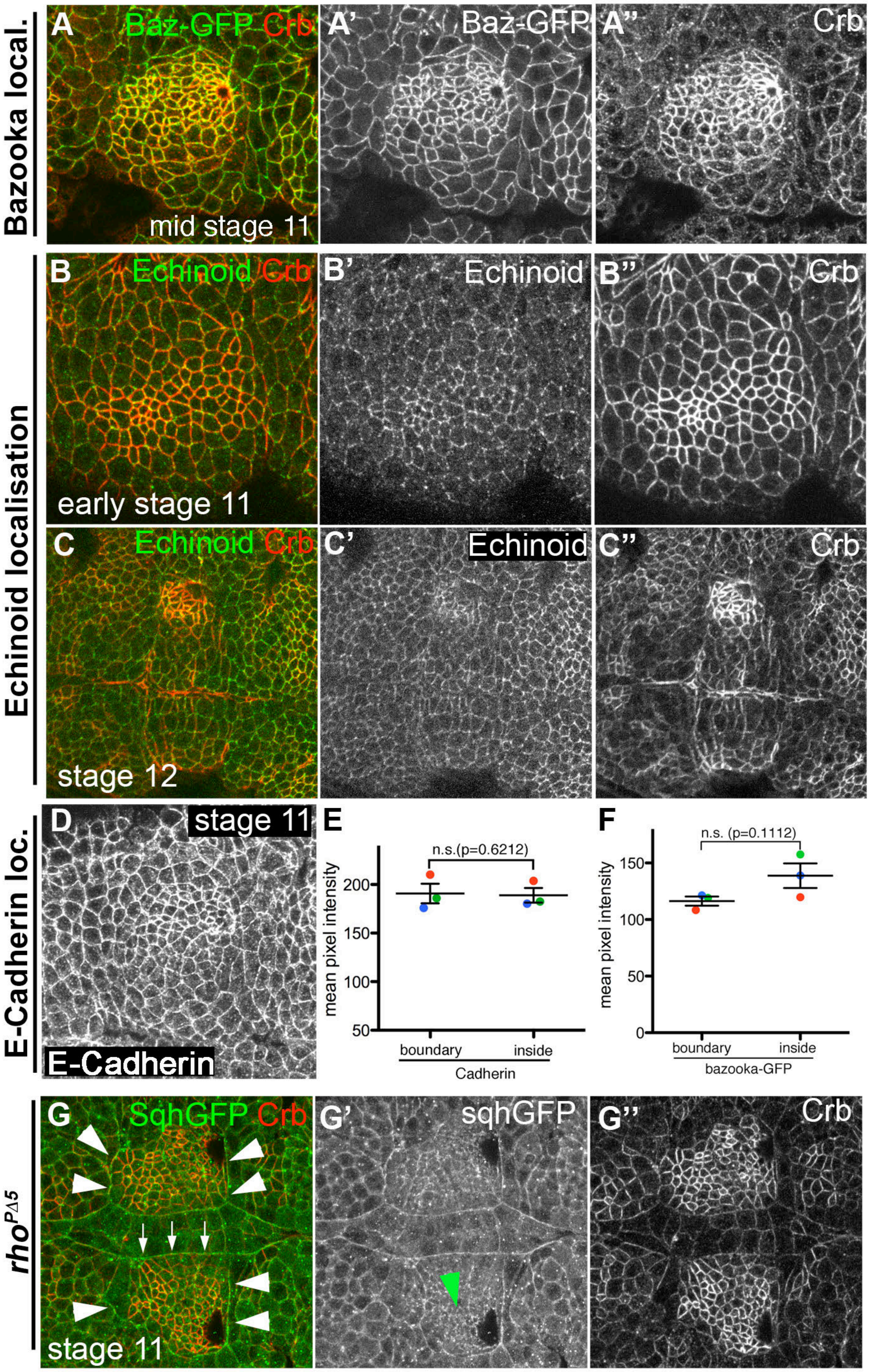

Röper Figure S2

Figure S2, related to Figure 4. Analysis of involvement of Bazooka/Par3, Echinoid, Cadherin, and EGFR signalling in actomyosin cable formation around the salivary gland placode.

A-A'' Confocal surface stack of an embryo carrying a GFP-exon trap in Bazooka (green **A,** and single in **A'**), labelled with Crumbs (red **A,** and single in **A''**) at stage 11. Note that Bazooka-GFP is not selectively depleted from the border of the placode in contrast to the situation e.g. at the DV-boundary of the wing disc (Major and Irvine, 2006). For quantification of Baz-GFP see **E** below.

B-C'' The homophilic cell adhesion molecule Echinoid is expressed throughout the embryonic epidermis during salivary gland invagination, but levels do not differ between placodal and surrounding cells. Echinoid (green in **B**, **C**, and single in **B'**, **C'**); Crumbs (red in **B**, **C**, and single in **B''**, **C''**).

D DE-Cadherin levels are not significantly different between the cell boundary where the actomyosin cable is located and the inside edges of these cells.

E,F Quantification of Cadherin (**E**) and Bazooka (**F**) mean pixel intensity in cell edges that form the boundary of the placode, or inside edges of the same cells (see schematic in Fig. 5E'). For both Cadherin and Bazooka there is no statistically significant difference in intensity. Shown is mean +/- SEM, data point colour indicates 'boundary/inside' measurement pairs from the same placode, p=0.6212 (n=3 pairs) for Cadherin, p=0.1112 (n=3 pairs) for Bazooka-GFP.

G-G'' In the absence of EGFR signalling, in a *rhomboid* null mutant ($rho^{P\Delta5}$), the actomyosin cable is formed properly. *sqhGFP* (green **E**, and single in **E'**), Crumbs (red **E**, and single in **E''**). Arrowheads in **E** point to the cable, arrows in **E** point to a much more pronounced cable on the ventral surface of the placode, likely induced by the fact that all placodal cells in the $rho^{P\Delta5}$ mutant show secretory cell identity and thus have highly upregulated levels of Crumbs. All cells at the boundary show strong Crumbs anisotropy. Note that cortical and medial myosin II are also present similar to the wild-type (arrow in **E'**).

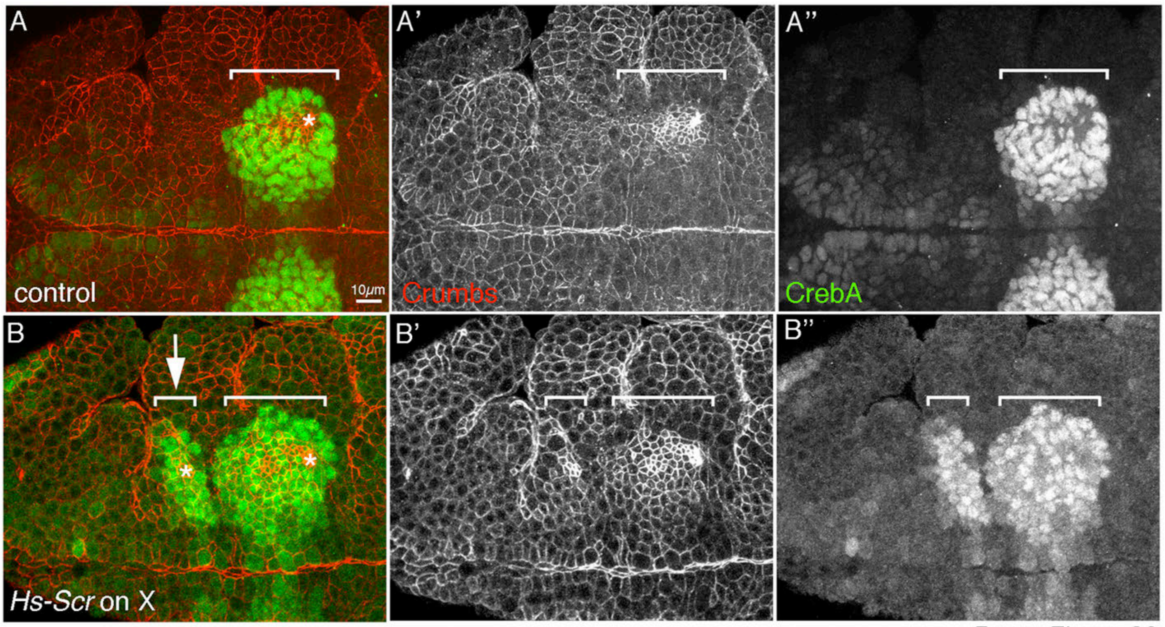

Röper Figure S3

Figure S3, related to Figure 5. Ectopic expression of Scr induces Crumbs accumulation and apical constriction.

Scr is overexpressed in embryos using a *hs-Scr* transgene. **A** Wild-type control, **B** example of a heat-shocked embryo. hs-induced overexpression of Scr leads to formation of an ectopic patch of CrebA expression anterior to parasegment 2 (placode area labeled by CrebA marked by large white bracket in all panels, ectopic patch of CrebA marked by a small white bracket; (Andrew et al., 1994; Henderson and Andrew, 2000). CrebA (green **A**,**B**, and single in **A''**, **B''**). Crumbs accumulation is induced in the patch of cells with ectopic CrebA expression (Crumbs, red in **A**, **B**, and single in **A'**, **B'**). Cells with high Crumbs levels show constricted apices and a shallow ectopic dimple (asterisks in **A**, **B** point to all invagination dimples observed in 3D-rendering, data not shown).

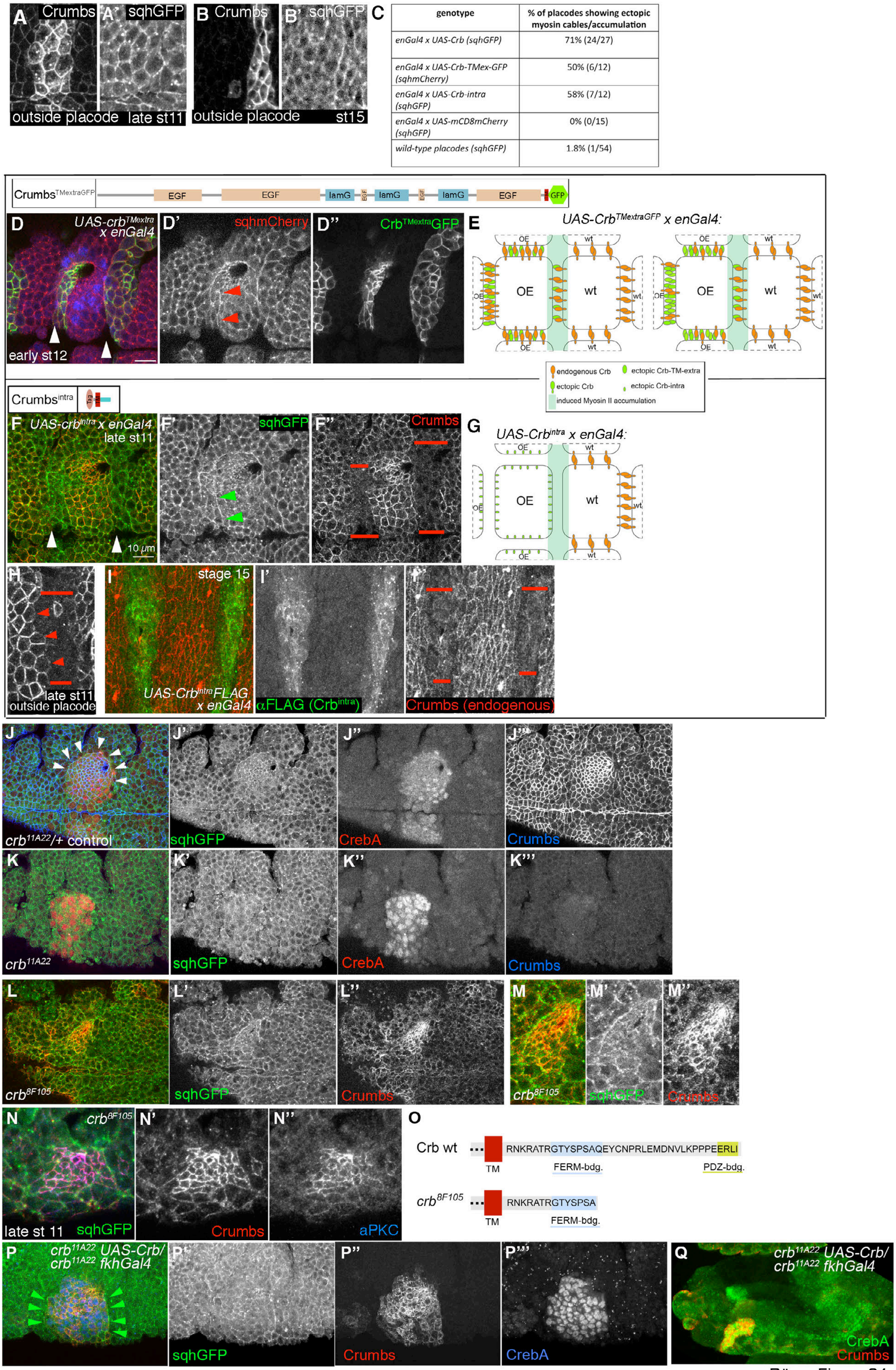

genotype	% of placodes showing ectopic myosin cables/accumulation
enGal4 x UAS-Crb (sqhGFP)	71% (24/27)
enGal4 x UAS-Crb-TMex-GFP (sqhmCherry)	50% (6/12)
enGal4 x UAS-Crb-intra (sqhGFP)	58% (7/12)
enGal4 x UAS-mCD8mCherry (sqhGFP)	0% (0/15)
wild-type placodes (sqhGFP)	1.8% (1/54)

Röper Figure S4

Figure S4, related to Figure 6. Analysis of overexpression of wild-type and deletion versions of Crumbs, and of myosin II localisation in *crb* mutants.

A-B' Ectopic Crumbs expressed in engrailed stripes in the epidermis posterior to the placode area: at early stages (11-12) no ectopic accumulation of myosin II is induced outside the placode (**A'**), whereas at later stages (15; **B'**) accumulation of myosin at the new border can occasionally be detected.

C Quantification of induction of ectopic myosin II accumulation and cable formation in different genetic backgrounds shown in Fig. 6 and this Figure S4.

D-E Overexpression of the extracellular and transmembrane domain of Crb (*UAS-*$Crb^{TMextra}$*,* shown schematically above the panels) induces formation of an ectopic myosin II cable within the placode (red arrowheads in **D'**) in half the embryos of this genotype. This is possibly due to the induction of a new border of high/low wild-type Crumbs accumulation. Myosin II is visualised using *sqh*::*sqh-mCherry* in this instance (red **D**, and single in **D'**); $\mathrm{Crb}^{\mathrm{TMextra}}$ (green **D**, and single in **D''**). **E** Schematics illustrating how overexpression of the transmembrane and extracellular domain of Crumbs under engrailed control could either lead to competition for binding between ectopic and endogenous Crumbs extracellular domains, thus also creating a new high/low boundary of endogenous Crumbs, which can induce ectopic accumulation of myosin II (top model), or could lead to downregulation of endogenous Crumbs, which would also titrate Crumbs intracellular tails away from the new boundary (lower model, see also Suppl. Fig. 5F-H'). **F-G** Overexpression of the transmembrane and intracellular domain of Crb (*UAS-*Crb^{intra}*,* shown schematically above the panels) induces a new border of high/low wild-type Crumbs accumulation through downregulation of endogenous Crumbs protein levels (Klebes and Knust, 2000) and leads to formation of an ectopic myosin II cable within the placode (green arrowheads in **F'**). Note that this construct induces the anisotropic re-distribution of wild-type Crumbs in cells that are bordering the overexpressing engrailed stripes (stripes marked by red lines in **F''**, **H**), both inside (**F''**) and outside (**H**) the placode. *SqhGFP* (green **F,** and single in **F'**), Crumbs (red **F**, and single in **F''**,**H**).

G Schematic illustrating how overexpression of the transmembrane and intracellular domain of Crumbs under engrailed control leads to drastic reduction in endogenous Crumbs protein levels in the overexpressing cells, thus creating a new high/low boundary of endogenous Crumbs, which can induce ectopic accumulation of myosin II. **I-I''** Overexpression of *UAS-Crb^{intra}* tagged with an extracellular FLAG-tag (Richard et al., 2009) in engrailed stripes does not reveal anisotropic localisation of Crb^{intra} at the border of expressing and non-expressing cells. Instead, Crb^{intra} localises all around the cell and also accumulates in intracellular vesicles. An example of an embryo at stage 15 is shown (visualised with an anti-FLAG antibody). *UAS-Crb^{intra}*-FLAG (green **I**, and single in **I'**), endogenous Crumbs (red in **I**, and single in **I''**). Red lines in **I''** indicate stripes of engrailed expression with reduced Crumbs^{wt} levels induced by the Crb^{intra} transgene expression.

J-K''' Comparison of s*qhGFP* localisation in a sibling control embryo (**J-J'''**) to a *crb* null mutant embryo, *crb^{11A22}* (**K-K'''**). In the mutant embryo overall levels of apical myosin II accumulation in the epidermal cells, including the placode, are reduced and no cable is visible (arrowheads in **J** mark the wild-type cable). No coordinated invagination or tissue bending occurs at early stages. **L-M''** In a hypomorphic *crb* mutant lacking only the last 23 intracellular amino acids (see schematic in panel B), the allele *crb^{8F105}*, the mutant Crumbs protein accumulates in the placode (**M''**), myosin II is enriched and localises at cortical and medial sites and as a cable surrounding the placode (**M'**). The epidermis in this mutant is disorganised, so the overall shapes of the placode and of the placodal cells are affected and invagination is not wild-type, but myosin II localisation and Crumbs anisotropy appear unaffected. **N-N''** In the *crb^{8F105}* allele, the truncated version of Crumbs that is expressed and lacks the PDZ-binding domain is still recruiting aPKC into an anisotropic localisation. *SqhGFP* (green **N**) , Crumbs (red **N,** and single in **N'**), aPKC (blue **N**, and single in **N''**). **O** Schematic illustrating the Crumbs intracellular tail of the wild-type protein (top) harbouring the membrane-proximal FERM-binding domain and the C-terminal ERLI/PDZ-binding motif. In *crb^{8F105}* the ERLI-motif is lost but the FERM-binding

domain is preserved (Medina et al., 2002). **P-P'''** Expression of wild-type Crumbs in a *crb* null embryo (*crb*^{11A22}) using *fkh-Gal4* leads to the occasional accumulation of myosin II and formation of segments of cables at the boundary of fkh expression (3 of 5 embryos at stage 11). In this situation, many glands that have nearly fully invaginated can be observed at stage 14 (**Q**; 87%, n=47), in contrast to *crb*^{11A22} where no invaginated glands with good morphology can be detected at this stage (0%, n=19). *sqhGFP* (green **P**, and single in **P'**); Crumbs (red **P**, **Q**, and single in **P''**); CrebA (to label gland identity, blue in **P**, and single in **P'''**, green in **Q**).

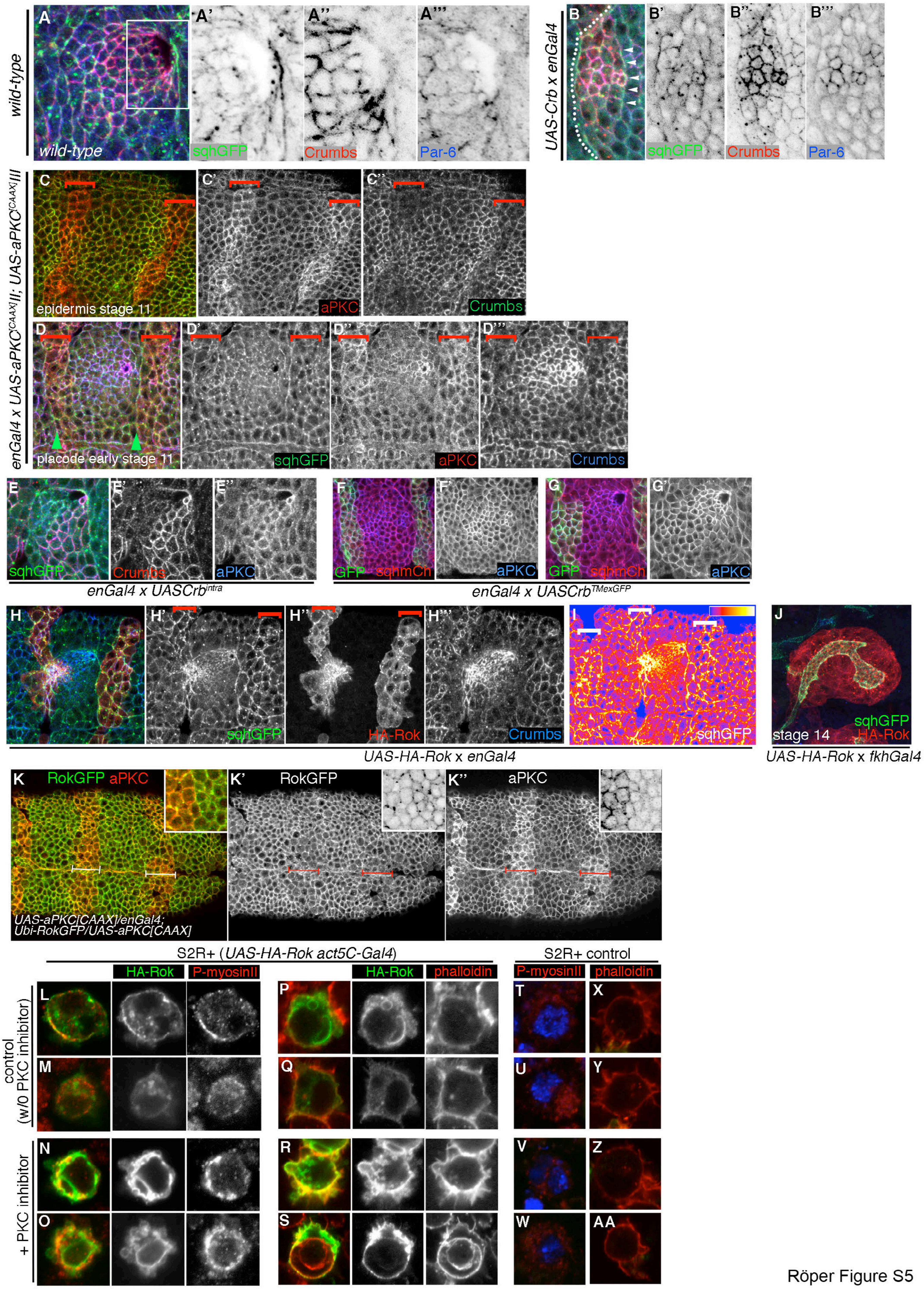

Röper Figure S5

Figure S5, related to Figure 7. Analysis of Par-6, aPKC and Rok as downstream effectors of Crumbs and upstream regulators of myosin II cortical association.

A-B''' High magnification analysis of Par-6 distribution at the boundary of a wild-type placode (**A-A'''**) and upon UAS-Crb expression in an engrailed stripe within the placode (**B-B'''**). The white box in **A** is the area magnified in **A'-A'''**, showing the posterior border of the wild-type placode. In contrast to Crumbs, Par-6 does not show anisotropic localisation at the boundary and is not complementary to myosin II accumulation. The dotted line in **B** indicates the anterior border of the placode, where the endogenous cable is located, whereas the arrowheads indicate the ectopic cable induced by Crumbs expression. *sqhGFP* (green in **A**, **B**, and single in **A'**, **B'**); Crumbs (red in **A**, **B**, and single in **A''**, **B''**); Par-6 (blue in **A**, **B**, and single in **A'''**, **B'''**).

C-D''' Expression of a membrane-targeted form of aPKC does not lead to aPKC anisotropic localisation or myosin cable induction *UAS-aPKC*$^{[CAAX]}$, a membrane targeted and thus active form of aPKC, was expressed under *en-Gal4* control. **C-C''** When aPKC$^{[CAAX]}$ overexpressing stripes are analysed within the epidermis at stage 11 (**C-C''**) or stage 14 (data not shown), the ectopic aPKC is not localised in an anisotropic fashion (red **C**, and single in **C'**). Indeed, ectopic aPKC$^{[CAAX]}$ negatively affects endogenous Crumbs levels (green **C**, and single in **C''**). **D-D'''** aPKC$^{[CAAX]}$ in a stripe within the salivary gland placode has the same effect: ectopic aPKC is not localised anisotropically (red **D**, and single in **D''**), a slight reduction of endogenous Crumbs is observed (blue **D**, and single in **D'''**), and no ectopic accumulation of myosin II is induced (green **D**, and single in **D'**). Red brackets indicate overexpression domains in all panels.

E-E'' Effect of expression of Crumbs^{intra} (using *enGal4*) on aPKC localisation within the placode: aPKC (blue and single in **E''**) largely follows Crumbs (red and single in **E'**) distribution and is depleted from the overexpression stripe, though some aPKC remains that might interact with diffuse Crumbs^{intra}. **F-G'** Effect of expression of Crumbs^{TMextraGFP} (using *enGal4*) on aPKC localisation within the placode: aPKC (blue

and single in **F’**, **G’**) is variable in the stripe expression domain, sometimes partially depleted (**F’**), sometimes mostly unaffected (**G’**) which could explain why ectopic myosin II accumulation is only observed in 50% of embryos.

H-H’’’ Expression of HA-Rok in enGal4 stripes leads to increased cortical association of myosin II/*sqhGFP* (red brackets in **H’** and **H’’** highlight the expression domain), and within the placode leads to constriction of apices. *SqhGFP* (green **H**, and single in **H’**), HA-Rok (red **H**, and single in **H’’**), Crumbs (blue **H**, and single in **H’’’**).

I The panel shows myosin II levels as a heat map (with dark blue representing lowest and white representing highest levels, see heat scale in top right corner of the panel). Note that within the en-Gal4 domains where Rok levels are increased (white brackets) an increase in cortical sqhGFP can be observed, consistent with a role of Rok in myosin cable assembly.

J Overexpression of *UAS-HA-Rok* within the whole salivary gland placode using *fkh-Gal4* leads to aberrant invagination and lumen defects at stage 14, indicating that Rok activity levels are tightly control, probably both temporally and spatially, during tube formation. *sqhGFP* (green); HA-Rok (red).

K-K’’ Expression of *UAS-aPKC[CAAX]* in enGal4 stripes leads to decreased cortical association of Rok-GFP and increased apical surface area. RokGFP (green in **K** and single in **K’**), aPKC (red in **K** and single in **K’’**). White and red brackets indicate overexpression domains. Insets show a boundary between wild-type and overexpressing cells.

L-AA Analysis of Rok-aPKC interactions in S2R+ cells. S2R+ cells expressing HA-Rok show membrane localisation of Rok (**L**,**M** and **P**, **Q**; 100%, n=326) and low levels of phospho-myosin II (**L**, **M**). Upon treatment with an aPKC inhibitory peptide, HA-Rok levels are strongly increased (**N**,**O**, **R**, **S**; 82%, n=170), as are levels of phospho-myosin II (**N**, **O**; 95%, n=59) and cortical actin (**R**, **S**; 100%, n=78) in these cells. Without ectopic HA-Rok expression there is no difference in phospho-myosin II or cortical actin levels (**T-AA**). All panels were scanned using identical settings.

Supplemental Experimental Procedures

Fly stocks and husbandry

The following transgenes were used: *sqh*^{AX3}*; sqh::sqhGFP42*, and *yw; sqhGFP40; sqhGFP40* (Royou et al., 2004), *sqh::sqhmCherry* (Martin et al., 2009), *enGal4*, *UAS-Crb(wt)* (Wodarz et al., 1995), *UAS-Crb-myc-intra* (Klebes and Knust, 2000) and *UAS-Crb-FLAG-intra* (Richard et al., 2009) [both kind gifts of Eli Knust], *UAS-Crb-TM extra-GFP* (Pellikka et al., 2002) ; kind gift of Uli Tepass), *hs-Scr* on X (Andrew et al., 1994; Henderson and Andrew, 2000) kind gift of Debbie Andrew, *bazookaGFP* (Buszczak et al., 2007), *UAS-aPKC[CAAX]* on II and III (Sotillos et al., 2004), *UAS-HA-Rok* (Wang and Riechmann, 2007) kind gift of Veit Riechmann, *Ubi-GFP-Rok* (unpublished; kind gift of Vincent Mirouse).

The following fly stocks were obtained from Bloomington or kindly donated by the indicated people and are described in FlyBase: *Scr*^{2} or *Scr*^{17} (amorphs); *crb*^{8F105} (Medina et al., 2002; Wodarz et al., 1993); *crb*^{11A22} (also called *crb*^{2}; null); *UAS-mCD8mCherry*.

To analyse myosin II localisation in mutants, virgins of *yw; sqhGFP4; sqhGFP40* were crossed to males of the mutant strains and the F1 backcrossed to each other. Mutant embryos of the following alleles were identified in the following ways: *Scr*^{2} or *Scr*^{17}: identified by lack of CrebA and other salivary gland markers; *crb*^{8F105}: hypomorphic allele with truncated protein remaining, identifiable by overall disorganised embryonic phenotype; *crb*^{11A22}: identified by lack of Crumbs.

Embryo Immunofluorescence Labelling, Confocal, Live and Super-resolution analysis

Embryos were collected on grape-juice plates and processed for immunofluorescence using standard procedures. Briefly, embryos were dechorionated in 50% bleach, fixed in 4% formaldehyde, and stained with phalloidin or primary and secondary antibodies in PBT (PBS plus 0.5% bovine serum albumin

and 0.3% Triton X-100). Crumbs, DE-Cadherin, and Engrailed antibodies were obtained from the Developmental Studies Hybridoma Bank at the University of Iowa; the anti-dCrebA antibody was a kind gift from Deborah Andrew (Andrew et al., 1997); the anti-Eyegone antibody was obtained from Silvia Aldaz (Aldaz et al., 2003); the anti-Echinoid antibody was obtained from Luis Escudero (Wei et al., 2005); the anti-Canoe antibody was obtained from Nick Brown (Takahashi et al., 1998); the anti-Stardust antibody was a gift from Eli Knust (Berger et al., 2007); anti-HA (Roche); anti phospho-myosin light chain 2 (S19) (Cell Signalling) . Secondary antibodies used were Alexa Fluor 488/Fluor 594/Fluor 649 coupled (Molecular Probes) and Cy3 and Cy5 coupled (Jackson ImmunoResearch Laboratories), and rhodamine-phalloidin was from Molecular Probes. Samples were embedded in Citifluor (Citifluor Ltd.) or Vectashield (Vectorlabs).

Images of fixed samples were acquired on an Olympus FluoView 1000 or a Zeiss 780 Confocal Laser scanning system as z-stacks to cover the whole apical surface of cells in the placode. Z-stack projections were assembled in ImageJ or Imaris (Bitplane), 3D rendering was performed in Imaris.

Tissue culture Transfection and Immunolabeling

S2R+ cells were transiently transfected using standard procedures. In brief, 3µg of DNA (UAS-vector plus act5cGal4 plasmid) were used to transfect near confluent cells using FugeneHD (Promega) following the manufacturer’s instructions. UAS-plasmids used were: pUAST-HA-Rok (kind gift of Veit Riechmann; (Wang and Riechmann, 2007)) and pUAST-Crumbs^{wt} (kind gift of Eli Knust; (Wodarz et al., 1995)). For immunolabelling cells were fixed in 4% formaldehyde, blocked with PBT (PBS +0.3% Triton X-100 and 0.5% BSA), and antibody incubations performed in PBT for 1hr in a humidified chamber. For inhibition of aPKC in S2R+ cells, cells were incubated after transfection overnight in the presence of 30mM PKCζ pseudosubstrate inhibitory peptide Myr-SIYRRGARRWRKL-OH (Calbiochem).

Smoothness quantifications

Similar to the definition of the straightness of a line (euclidian distance between points A and B divided by actual length of line between A and B, with a perfect straight line having a straightness of $s=1$, Fig.6H), the smoothness of a curve can be defined as the euclidian distance between points A and B divided by the actual distance of the line (in our case boundary measured at the level of apical membrane edges; Fig.6H). A smooth curve has a value of smoothness of $s\sim0.9$, whereas an edgy curve will have a value $s\ll0.9$.

Lengths of representative boundaries (using Crumbs or Cadherin labeling to identify the apical membrane edges) were measured in Image J, plotted in Prism and statistical significance of differences in smoothness between different genetic samples analysed using unpaired t-test.

Supplemental References

Andrew, D.J., Baig, A., Bhanot, P., Smolik, S.M., and Henderson, K.D. (1997). The Drosophila dCREB-A gene is required for dorsal/ventral patterning of the larval cuticle. Development *124*, 181-193.

Berger, S., Bulgakova, N.A., Grawe, F., Johnson, K., and Knust, E. (2007). Unraveling the genetic complexity of Drosophila stardust during photoreceptor morphogenesis and prevention of light-induced degeneration. Genetics *176*, 2189-2200.

Buszczak, M., Paterno, S., Lighthouse, D., Bachman, J., Planck, J., Owen, S., Skora, A.D., Nystul, T.G., Ohlstein, B., Allen, A.*, et al.* (2007). The carnegie protein trap library: a versatile tool for Drosophila developmental studies. Genetics *175*, 1505-1531.

Takahashi, K., Matsuo, T., Katsube, T., Ueda, R., and Yamamoto, D. (1998). Direct binding between two PDZ domain proteins Canoe and ZO-1 and their roles in regulation of the jun N-terminal kinase pathway in Drosophila morphogenesis. Mech Dev *78*, 97-111.

Wang, Y., and Riechmann, V. (2007). The role of the actomyosin cytoskeleton in coordination of tissue growth during Drosophila oogenesis. Curr Biol *17*, 1349-1355.

Wei, S.Y., Escudero, L.M., Yu, F., Chang, L.H., Chen, L.Y., Ho, Y.H., Lin, C.M., Chou, C.S., Chia, W., Modolell, J.*, et al.* (2005). Echinoid is a component of adherens junctions that cooperates with DE-Cadherin to mediate cell adhesion. Dev Cell *8*, 493-504.

Wodarz, A., Grawe, F., and Knust, E. (1993). CRUMBS is involved in the control of apical protein targeting during Drosophila epithelial development. Mech Dev *44*, 175-187.
