# Supplementary figures and images for: Anisotropy of Crumbs and aPKC Drives Myosin Cable Assembly during Tube Formation
Source: Dev Cell. 2012 Nov 13;23(5):939–53. doi: 10.1016/j.devcel.2012.09.013 (PMC3562440; doi:10.1016/j.devcel.2012.09.013)

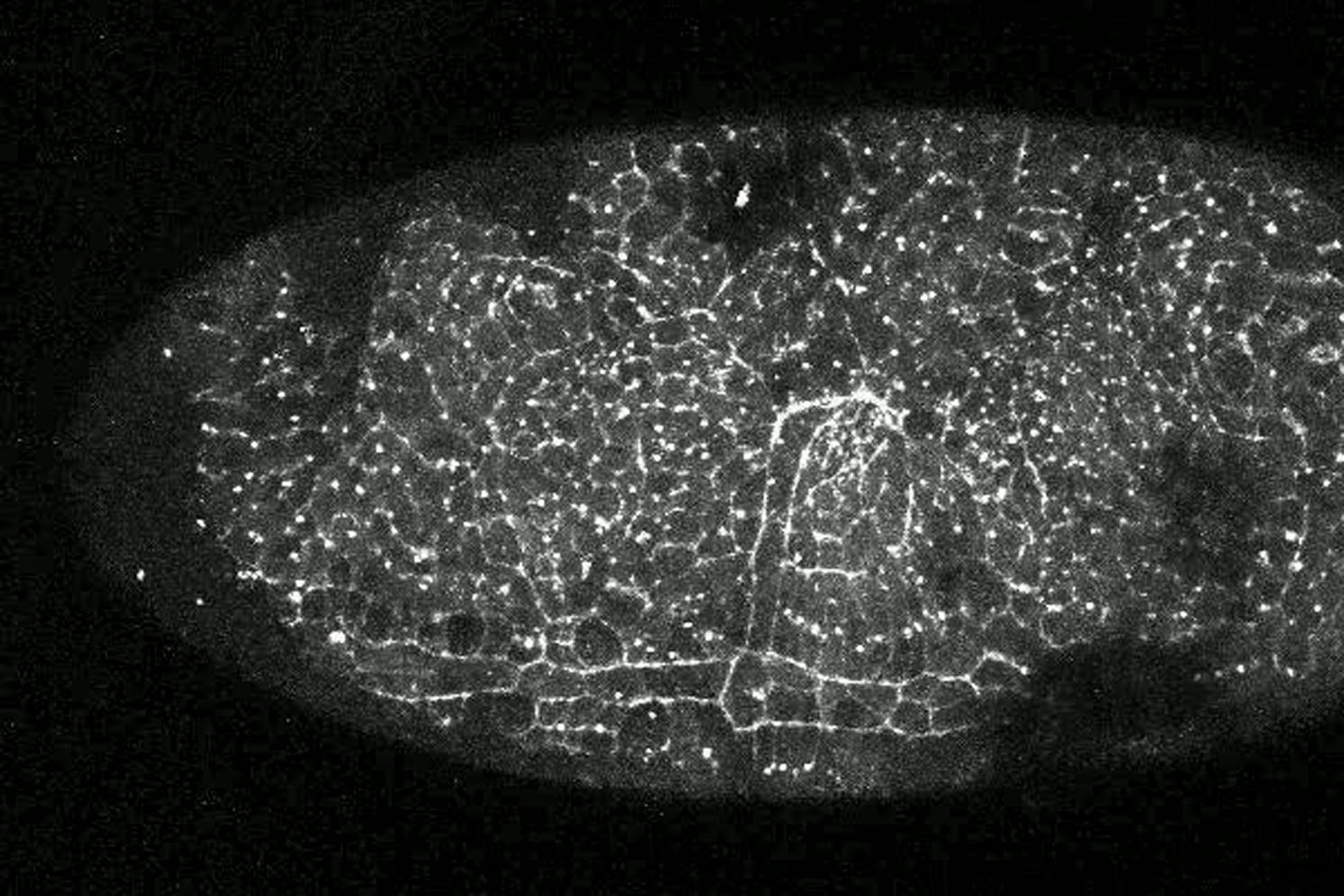

Supplement: Movie S1. A Prominent Myosin II Cable Surrounds the Salivary Gland Placode during Tube Formation, Related to Figure 1 — Time-lapse movie of a sqhAX3; sqh::sqhGFP42 embryo, viewing the area around one placode on the ventral side of the embryo. A still of this movie is shown in Figure 1. The movie is a projection of a confocal z stack to cover all apical myosin II in the area imaged. Length of the movie is 54 min; z stacks were acquired every 3 min. [file mmc2.jpg]

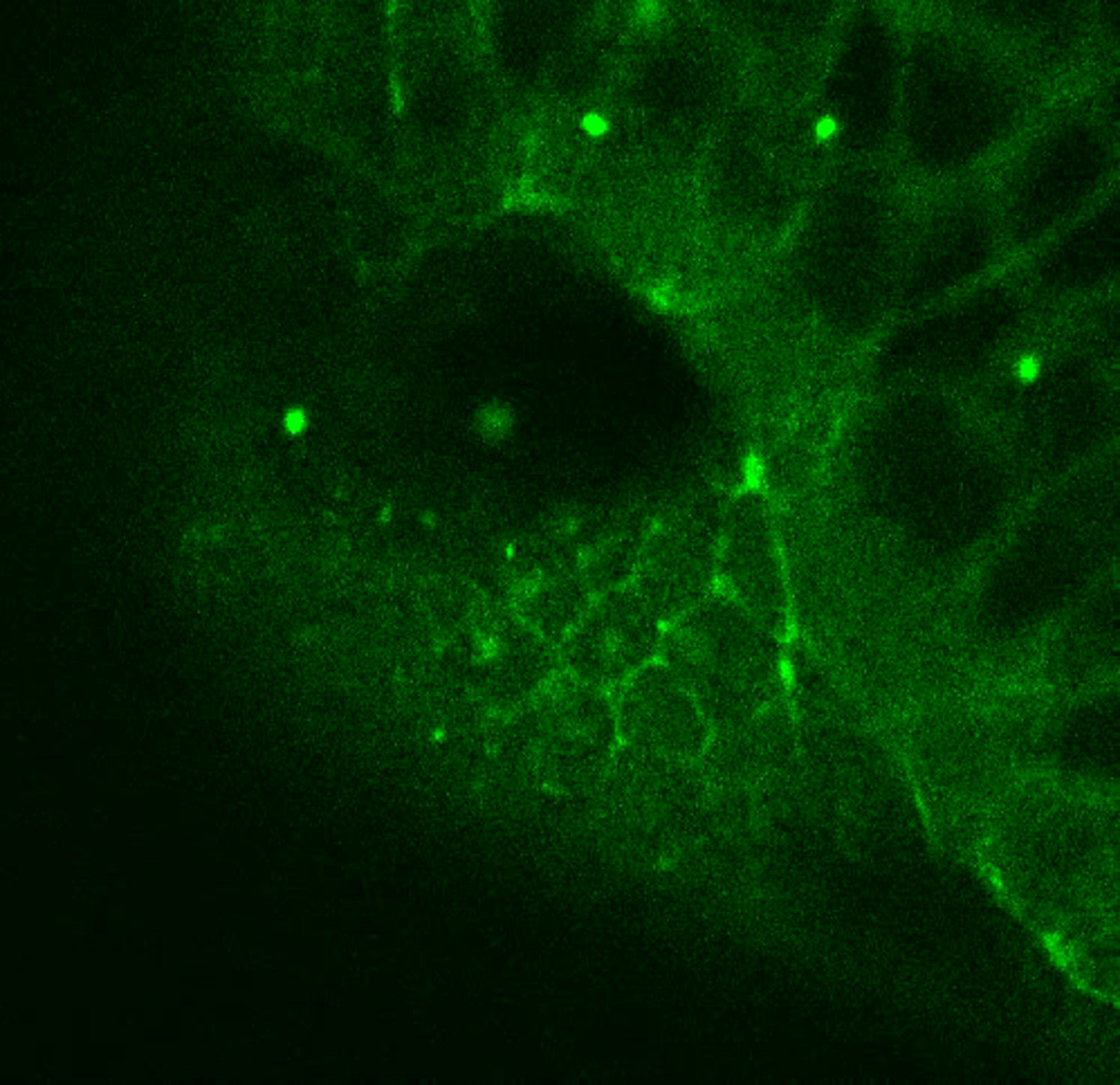

Supplement: Movie S2. Laser Ablation of a Cell Edge that Was Part of the Myosin Cable around the Salivary Gland Placode, Related to Figure 2 — Time-lapse movie of sqhGFP; a single confocal section is shown. Frames are ∼1.2 s apart; the cable is ablated after time point 5 (green arrow appearing to indicate the position of ablation). [file mmc3.jpg]

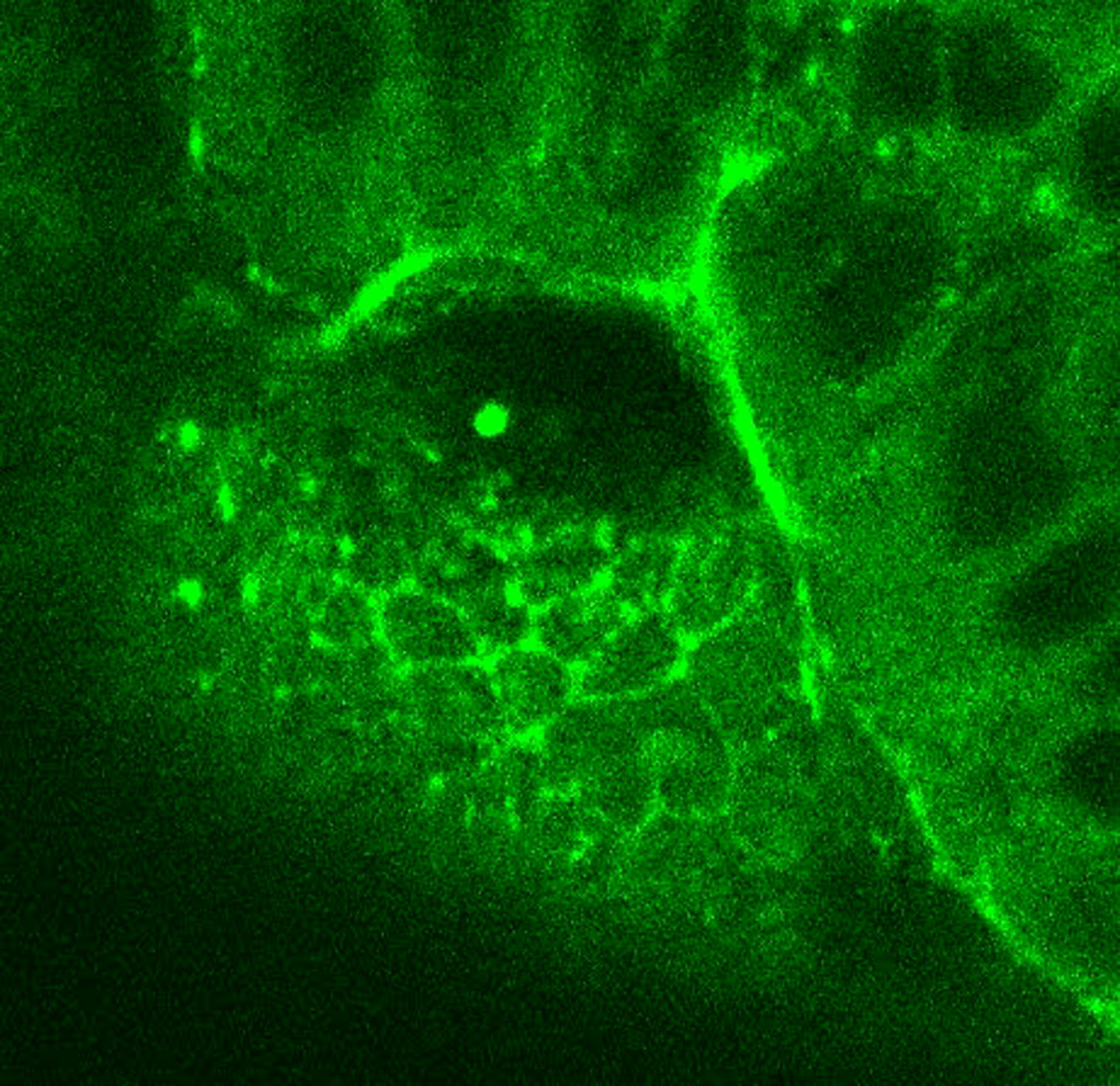

Supplement: Movie S3. Laser Ablation of a Control Cell Edge Enriched in Myosin, Related to Figure 2 — Time-lapse movie of sqhGFP; a single confocal section is shown. Frames are ∼1.2 s apart; a myosin-rich edge within the placode that is not part of the myosin cable is ablated after time point 5 (green arrow appearing to indicate the position of ablation). [file mmc4.jpg]

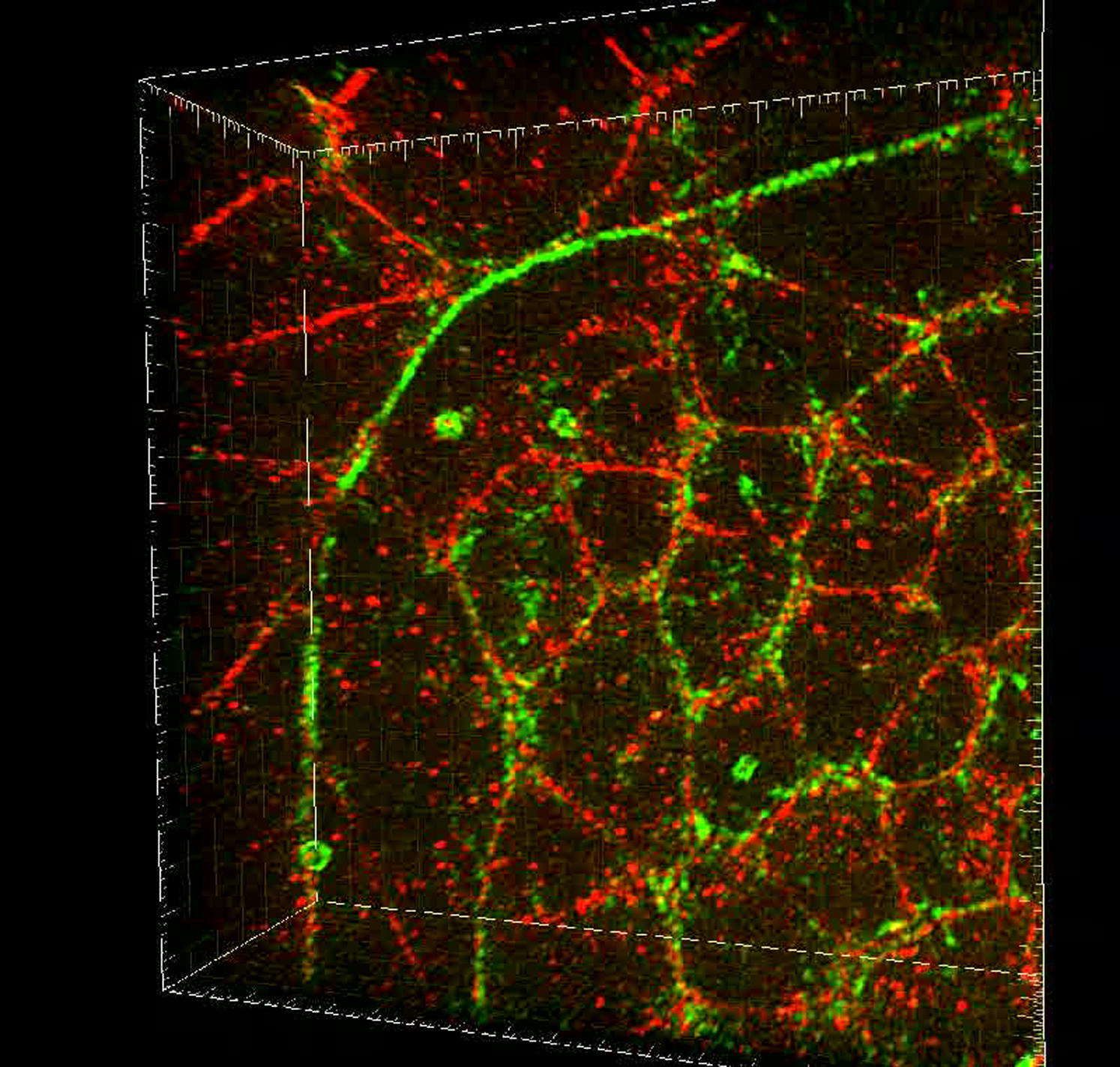

Supplement: Movie S4. Animated 3D-Volume Rendering of a SI-M Stack Comparing sqhGFP and DE-Cadherin, Related to Figure 3 — sqhGFP is in green and DE-cadherin in red. Scale bar is 2 μm, and the depth of the stack is 6 μm. Note that the bright doughnut-shaped structures strongly labeled by sqhGFP are remnants of cleavage furrows that accumulate ectopically in the sqhAX3;sqh::sqhGFP42 transgenic embryos but that appear to have no effect on sqhGFP function in this genetic background (Royou et al., 2004). [file mmc5.jpg]
